# The Natural Products Targeting on Allergic Rhinitis: From Traditional Medicine to Modern Drug Discovery

**DOI:** 10.3390/antiox10101524

**Published:** 2021-09-26

**Authors:** Suhyun Lim, Iwah Jeong, Jonghyeok Cho, Chaewon Shin, Kwan-Il Kim, Bum-Sang Shim, Seong-Gyu Ko, Bonglee Kim

**Affiliations:** 1 College of Korean Medicine, Kyung Hee University, Seoul 02447, Korea; lawinno@khu.ac.kr (S.L.); iwah@khu.ac.kr (I.J.); dkfmaekdnjfk@khu.ac.kr (J.C.); name1488@khu.ac.kr (C.S.); eshimbs@khu.ac.kr (B.-S.S.); epiko@khu.ac.kr (S.-G.K.); 2 Division of Allergy, Immune and Respiratory System, Department of Internal Korean Medicine, Kyung Hee University, Seoul 02447, Korea; myhappy78@naver.com; 3 Korean Medicine-Based Drug Repositioning Cancer Research Center, College of Korean Medicine, Kyung Hee University, Seoul 02447, Korea

**Keywords:** allergic rhinitis, natural products, antioxidants, inflammation, interleukin, nuclear factor kappa-light-chain-enhancer of activated B cells

## Abstract

More than 500 million people suffer from allergic rhinitis (AR) in the world. Current treatments include oral antihistamines and intranasal corticosteroids; however, they often cause side effects and are unsuitable for long-term exposure. Natural products could work as a feasible alternative, and this study aimed to review the efficacies and mechanisms of natural substances in AR therapies by examining previous literature. Fifty-seven studies were collected and classified into plants, fungi, and minerals decoction; clinical trials were organized separately. The majority of the natural products showed their efficacies by two mechanisms: anti-inflammation regulating diverse mediators and anti-oxidation controlling the activity of nuclear factor kappa-light-chain-enhancer of activated B cells (NFκB) pathway stimulated by reactive oxygen species (ROS). The main AR factors modified by natural products included interleukin (IL)-4, IL-5, IL-13, interferon-gamma (IFN-γ), tumor necrosis factor-α (TNF-α), cyclooxygenase 2 (COX-2), and phospho-ERK1/2 (p-ERK1/2). Although further studies are required to verify their efficacies and safeties, natural products can significantly contribute to the treatment of AR.

## 1. Introduction

Allergic rhinitis (AR), also known as hay fever, is inflammation inside the nose stimulated by specific allergens, usually pollen or dust [1]. Due to its high prevalence and the negative impact on patients’ quality of life, AR is regarded as a major chronic disease. More than 500 million people suffer from AR, and up to 40% of the global population is affected [2]. It is an immunoglobulin E (IgE)-mediated disorder in the nose and triggered by the interaction between specific mast cell IgE antibodies and allergens present in the air. The interaction causes the mast cells to release chemicals that act as inflammatory agents, thus resulting in inflamed marginal tissues [3]. Consequently, it is considered part of a systemic inflammatory process. The symptoms include rhinorrhea, nasal itching, sneezing, and nasal congestion, and are also associated with upper and lower airway membrane disorders: asthma, rhinosinusitis, conjunctivitis, and polyposis [4].

Histamine released from mast cells is the dominant factor of allergic reactions, and the secretion of interleukin (IL)-4 and IL-5 follows, generating T helper cell 2 (Th2) cytokine response. It is, therefore, crucial to control histamine pathways in treating AR. Current therapeutic interventions commonly used are oral antihistamines and intranasal corticosteroids [5]. Antihistamines block the binding of histamine to the H1 receptor, preventing the release of histamines [6]. Although second-generation antihistamines have fewer side effects, they are still sedating and may provoke psychomotor retardation and reduced academic performance [7,8]. Intranasal corticosteroids control inflammation by regulating mediator release [9]. However, long-term exposure may increase the risk of osteoporosis, fractures, cataracts, hyperglycemia, infection risk, slower wound healing, and headache [10]. Natural products could be plausible options for curing AR, as treatments that cover these adversaries and effectively maintain therapeutic efficacy are in need.

Plant extracts have been used as conventional treatment for thousands of years in many countries, including Korea, Japan, China, etc. [11,12]. Unlike synthetic drugs, they down-regulate harmful side effects and mitigate the damages of various therapy, such as onco-chemotherapy or radiotherapy [13,14]. Furthermore, they are capable of multi-targeting the immune system since they consist of diverse molecules in nature. Many of the previous works of literature have demonstrated the individual efficacy of each product in treating allergic symptoms, but an overall review of these plant extracts is absent. This paper concentrated on plant extracts that have been tested as a remedy for allergic rhinitis and examined their mechanisms, aiming to invigorate the use of natural substances in AR therapies.

## 2. Pathogenesis of Allergic Rhinitis

### 2.1. T Helper Cell Differentiation

**Figure 1:**
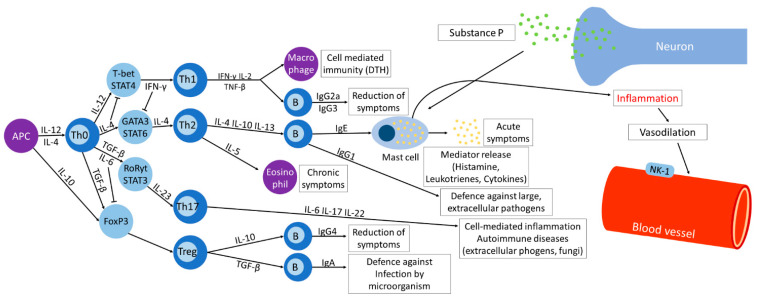
The mechanism of allergic rhinitis (AR) symptoms triggered by APC. APC, antigen-presenting cell; IL, interleukin; Th, T helper; T-bet, T-box-expressed-in-T-cells; STAT, signal transducers and activators of transcription; TGF, transforming growth factor; RoRyt, retinoid-related orphan receptor; FoxP3, Forkhead box p3; IFN, interferon; Treg, regulatory T; DTH, delayed type hypersensitivity; Ig, immunoglobulin; NK-1, neurokinin-1 receptor.

There are several mechanisms of AR; Th cell differentiation can primarily lead to AR symptoms (Figure 1). Allergens trigger IgE to bind to the high-affinity receptor on the surface of mast cells, and diverse mediators such as histamine, leukotrienes, and cytokines are subsequently released [15,16,17,18,19]. These agents create inflammation mostly in the nasal mucosa, causing mucus secretion, sneezing and sometimes reduce the size of airways by inducing vasodilation of blood vessels. Several mechanisms produce immune responses, thus leading to allergic reactions. IL-4 and IL-12 from the antigen-presenting cell (APC) initiate the manipulation of Th cells. Th cells differentiate from Th0 cells, a common precursor, into Th1, Th2, Th17, or regulatory T (Treg) cells. For Th1 cells to differentiate from Th0 cells, IL-12 activates T-box-expressed-in-T-cells (T-bet) through STAT4 [20]. Th1 cells immunize against pathogens invading into other cells by secreting mainly IL-2, IFN-γ, and TNF-α. Cytokines secreted by Th1, IL-2, IFN-γ, and transforming growth factor (TGF)-β, elicit immunoglobulin G2a (IgG2a) that reduces symptoms. IFN-γ is also responsible for cell-mediated immunity by instigating a macrophagic reaction, hence generating delayed-type hypersensitivity (DTH). IL-4 stimulates the activation of GATA binding protein 3 (GATA3) and signal transducer and activator of transcription 6 (STAT6), the factors related to Th2 cells differentiation, while suppressing T-bet and STAT4. Th2 cells produce IL-4, IL-10, and IL-13 for IgE and IgG1 promotion and IL-5 for eosinophil growth and differentiation. IgE production by B cells is followed by the release of the mediator, histamine, leukotrienes, prostaglandins, and cytokines, by mast cells. The products elicit acute symptoms, whereas the eosinophil is responsible for chronic symptoms. IgG1 from B cells is involved in defense against huge, extracellular pathogens. Substance P (SP) binding to the neurokinin-1 receptor (NK-1R) leads to vasodilation and the release of histamine from mast cells [21,22,23]. TGF-β and IL-6 together regulate STAT3 and retinoid-related orphan receptors (RoRyt). RoRyt secretes IL-23, which initiates the alteration of Th0 into Th17 cells. Th17 cells are accountable for cell-mediated inflammation and autoimmune diseases induced while protecting the host against extracellular pathogens and fungi. Lastly, Th0 cells are transited to Treg cells by activation of Forkhead box p3 (Foxp3). Treg cells secrete IL-10 and TGF-β, acting on B cells to produce IgG4 and IgA, respectively. IgG4 reduces symptoms, while IgA defends against infection by microorganisms.

### 2.2. Histamine Receptors

Histamine is released when allergens induce nasal mast cell degranulation, thus releasing mediators into the nasal mucosa [24]. Histamine, converted from histidine by histidine decarboxylase (HDC), mediates allergy and inflammation by activating histamine receptors [25,26,27,28]. There are four histamine receptors, but only three are known for their separate roles, mainly conducting the early phase of allergen responses whose symptoms fade within 15–20 min. The histamine H1 receptor (H_1_R) induces symptoms, such as sneezing, pruritus, rhinorrhea, and nasal blockage by activating nasal nociceptive sensory nerves (5th nerve). The stimulation of sensory nerves causes the release of acetylcholine from nasal parasympathetic nerves and central reflex-dependent bilateral increase, resulting in increased nasal mucus secretion. H_1_R coupled with G protein G_q/11_ activates phospholipase C (PLC), stimulating 1, 4, 5-inositol trisphosphate (IP_3_) production to induce Ca^2+^ mobilization and diacylglycerol (DAG), which activates protein kinase C (PKC) [29,30]. PKC activates the transcription factor NF-κB, resulting in augmented transcription of proinflammatory genes. Ca^2+^ promotes the vasodilation of nasal blood vessels as well [31]. The histamine H2 receptor (H_2_R), coupled with G_S_-proteins, mainly leads to nasal blockage. H_2_R coupled with G_S_-proteins activates adenylyl cyclase (AC). AC stimulates cyclic adenosine monophosphate (cAMP) production, inducing cAMP-responsive element-binding protein (CREB) [32,33,34]. cAMP-dependent protein kinase (PKA) signaling inhibits leukotriene synthesis in neutrophils [35]. The histamine H3 receptor (H_3_R) suppresses substance P release from nasal nociceptive sensory nerves. H_3_R coupled with G_i/O_-protein negatively regulates AC; however, it activates mitogen-activated protein kinase (MAPK) and phosphatidylinositol 3-kinase (Pl3K), which stimulates the phosphorylation of Akt [36,37,38]. H_3_R also promotes IgE-mediated activation of mast cells degranulation [39].

### 2.3. 5-Lipooxygenase Pathway and Prostaglandin E2 Synthesis-and-Signaling Pathway

The 5-lipooxygenase (LO) pathway and prostaglandin E2 (PGE_2_) synthesis-and-signaling pathway are related to AR. ROS stimulates the activity of mitogen-activated protein kinase (MAPK) and nuclear factor kappa-light-chain-enhancer of activated B cells (NF-κB) pathway, thus inducing the expression of COX-2 [40,41]. As a result, PGE_2_ signals occur through EP_1_, EP_2_, EP_3_, and EP_4_ [42]. Activation of G protein-coupled receptors EP_1_-EP_4_ through binding with PGE_2_ leads to the regulation of intracellular Ca^2+^ and cAMP. Activated EP_1_ and EP_3_ increase intracellular Ca^2+^, EP_2_ and EP_4_ provoke the cAMP production, while EP_3_ suppresses it [43]. The dinitrophenyl (DNP) antigen activates immunoreceptor tyrosine activation motifs (ITAM)-Spleen tyrosine kinase (SyK) pathway by binding to the IgE anti-DNP/FcεRI receptor complex, resulting in degranulation by the activation of PKC [44]. Activated PKC induces the activation of phospholipase A_2_, increasing arachidonic acid (AA) bioavailability related to metabolism [45]. AA is converted into 5-hydroperoxy eicosatetraenoic acid (5HPETE), then leukotriene A_4_ (LTA_4_), and leukotriene C4 (LTC_4_) subsequently by 5-lipoxygenase (5-Lox). The increase in intracellular calcium results in degranulation. The conversion of AA to 5HPETE, then to LTA_4_, is stimulated by 5-Lox and 5-LO-activating protein (FLAP) [46,47]. LTA_4_ produces LTC_4_, LTD_4_, and LTE_4_ or transforms into LTB_4_. LTC_4_, LTD_4_, and LTE_4_ bind to cysteinyl leukotriene 1 (CystLT1) receptors, evoking chemotaxis, chemokine expression, and EC proliferation [48,49].

### 2.4. Immune Response Pathways

Src family tyrosine kinase Lyn activates kinase signaling in B lymphocytes, while caspase-1 mediates secretion of proinflammatory cytokines, resulting in immune responses. B cell receptor (BCR) is composed of membrane-bound Ig and heterodimeric signaling subunit Ig-α/Ig-β [50,51]. When the antigen binds with BCR, Ig-α/β ITAMs are activated and phosphorylated by Lyn, resulting in the activation of Syk. Activated Syk phosphorylates activate Bruton’s tyrosine kinase (BTK) and phospholipase C gamma 2 (PLCγ2). Recruitment of BTK follows. Syk also induces the recruitment and phosphorylation of p85, resulting in the generation of phosphatidylinositol 3, 4, 5-trisphosphate (PIP_3_) from phosphatidylinositol 4, 5-trisphosphate (PIP_2_). PIP_3_ phosphorylates Akt, activating alpha and beta subunits of the IκB kinase (IKK β/α), nuclear factor of kappa light polypeptide gene enhancer in B-cells inhibitor alpha (IκBα), and the p65 NF-κB are phosphorylated by IKK β/α, then degraded and translocated, respectively. Lyn, Syk, and BTK stimulate the phosphorylation and activation of PLCγ2, which hydrolyzes PIP_2_, generating inositol 3, 4, 5-triphosphate (IP_3_) and DAG. Ca^2+^ mobilization and PKC-β stimulation results in MAPK pathway activation, NF-κB, and the nuclear factor of activated T-cells (NF-AT) nuclear location. Through IL-1β signaling, PKC δ activates NF-κB [52,53]. Pro-caspase-1, apoptosis-associated speck-like protein containing a CARD (ASC), and NLR family pyrin domain containing 3 (NLRP3) are combined to form a complex which activates caspase-1, a factor that activates and releases IL-1β and IL-18 [54].

## 3. Natural Products That Inhibit Allergic Rhinitis

### 3.1. Plant-Originated Natural Products

Plant-originated natural products are substances derived from herbs, flowers, trees, etc. They are naturally extracted rather than artificially synthesized. The efficacy of plants containing medical substances is principally descendent from the past, and sometimes they are newly discovered.

#### 3.1.1. In Vitro Studies

There were eighteen in vitro studies of plant-originated natural products (Table 1). The bark of *Albizia lebbeck* is an ingredient of Ayurvedic Kadha, which has been used for treating asthma for more than two thousand years [55]. Propidium Monoazide-treated HeLa cells were administered with 0.1, 1, and 10 μg/mL of *Albizia lebbeck* bark ethanol extract for 24 h. Additionally, histamine-treated HeLa cells were administered with 10 μg/mL of the same extract for 24 h. Both treatments were related to the degradation of IL-4, IL-5, IL-13, H1R, and HDC.

Brazilian green propolis (BGPP), mainly isolated from *Baccharis dracunculifolia* and produced in Southeast Brazil, has been extensively consumed as a dietary supplement. HeLa cells were administered with 25, 75, 100, 125, and 200 µg/mL of BGPP ethanol extract for 3 h [56]. Additionally, RBL-2H3 cells were treated with 25, 75, and 100 µg/mL of BGPP ethanol extract for 3 h. The first experiment degraded levels of H1R mRNA, p-PKCδ, and IL-9, and the second decreased levels of IL-9.

Tani et al. demonstrated Brazilian green propolis ethanol extract prevented nasal obstruction through degradation of IL-5, IL-14, and Cysteinyl leukotrienes (CysLTs) [57]. Cry j1-treated peripheral blood mononuclear cells (PBMCs) were treated with 3, 10, and 30 μg/mL of Brazilian green propolis extract for 6 days.

Chrysin (5, 7-dihydroxyflavone) is isolated from propolis, blue passionflower (*Passiflora caerulea*), vegetables, and fruits [58]. Several studies have reported that chrysin has beneficial effects, including anti-tumor and antioxidant activities. Anti-DNP IgE-sensitized RBL-2H3 cells, phorbol myristate acetate (PMA)/A23187-treated RBL-2H3 cells, and PMA/A23187-treated HMC-1 were administered with 0.1, 1, and 10 µg/mL of Chrysin for 30 min. Chrysin inhibited the mast cell-derived allergic inflammatory reaction by decreasing TNF-α, IL-1β, IL-4, IL-6, caspase-1, and NF-κB levels.

A combination of preparations from Citrus and Cydonia have been commonly used as injections or sprays to treat allergic patients during the past hundred years [59]. *Citrus limon* Burm. f. was stimulated on PBMCs at the amount of 0.01 g/mL for 7 days [60]. PBMCs were obtained from the blood of five grass pollen-allergic donors aged 18–40 and five healthy eligible participants who had no sign of allergic rhinitis. Citrus reduced Th2 pathway activation by ameliorating the level of IFN-γ and down-regulating TNF-α, IL-5 cytokines. By using the same method, *Cydonia oblonga* Mill. was stimulated. Cydonia was mainly concerned with increasing the innate related Th1 pathway activity by the induction of the IFN-γ cytokine.

*Elsholtzia ciliata* (Thunb.) Hyl. is a herb that has been used as a traditional oriental medication for the treatment of fever, headache, diarrhea, and edema [61]. A water extract of *Elsholtzia ciliata* (Thunb.) Hyl. (Labiatae) inhibited mast cell-mediated allergic reactions and treated allergic inflammatory disease. PMACI exposed HMC-1 were treated with 1, 10, and 100 μg/mL of water extract of Elsholtzia ciliate for 30 min. TNF-α, IL-6, IκBα, NF-κB, and phospho-p38 mitogen-activated protein kinases (p-p38 MAPK) were degraded.

Shaha et al. injected 25, 75, 100, 125, and 200 µg/mL of enzyme-treated (protease-degraded) royal jelly (3.5% (E)-10-hydroxy-2-decenoic acid and 0.6% 10-hydroxydecanoic acid) to HeLa cells and RBL-2H3 cells for 12 h [56]. Decreased levels of H1R mRNA, p-PKCδ, and IL-9 were confirmed, suggesting that royal jelly improved allergic nasal symptoms.

Magnoliae Flos is an oriental herb commonly used in traditional Chinese and Korean medicine formulations [62]. It has been commonly used as a symptomatic relief to allergic rhinitis, sinusitis, and headache. Kim et al. administered 0.1, 0.3, and 1 mg/mL of FM ethanol extract (FM EtOH) to hANO1-transfected HEK293T cells for 600 s. Additionally, HEK293T and Calu-3 cells were treated with 30, 100, and 300 μg/mL of FM EtOH for 600 s.

Gencydo^®^, a combination of lemon (*Citrus limon*) juice and aqueous quince (*Cydonia oblonga*) extract has been used traditionally in anthroposophical medicine for treating patients with allergic rhinitis or asthma [63]. DNP-HSA-treated RBL-2H3 were treated with 0.2, 0.4, and 0.8 mg/mL of Genydo^®^ for 10 min, thus inhibiting the release of histamine and degranulation. Meanwhile, TNF-α/IL-4-exposed human bronchial epithelial cells, PMA/A23187-activated HMC, and granulocyte-macrophage colony-stimulating factor (GM-CSF)-treated human eosinophil granulocytes were treated with 0.2, 0.4, and 0.8 mg/mL of Gencydo^®^ for 24 h. Gencydo^®^ inhibited the release of eotaxin and restrained early and late-phase allergic reactions when injected into human bronchial epithelial cells and HMC, respectively. However, Gencydo^®^ had no impact on the state of GM-CSF-treated human eosinophils.

*Lindera obtusiloba*, a traditional Korean medicine, has been used in the treatment of inflammation and improvement of blood circulation [64]. Herbal infusions of *Lindera obtusiloba* have been used to treat chronic liver disease. PMACI-treated HMC-1, dinitrophenyl-human serum albumin (DNP-HSA)-treated RBL-2H3, and DNP-HSA-treated RPMCs were administered with 0.1, 1, 10, and 100 μg/mL of *Lindera obtusiloba* water extract for 30 min. *Lindera obtusiloba* water extract reduced histamine release and inhibited systemic and local allergic reactions.

Ortho-vanillic acid (2-hydroxy-3-methoxybenzoic acid), isolated from *Amomum xanthioides,* strongly inhibited mast cell-mediated allergic inflammation [65]. RPMCs and ortho-vanillic acid-treated RBL-2H3 cells were injected with 1, 10, and 100 nmol/L of ortho-vanillic acid for 24 h. Degradation of TNF-α, IL-4, phospho-Lyn (p-Lyn), p-Syk, and p-Akt was involved.

The roots of *Ostericum koreanum* Maxim. (Osterici Radix) have been traditionally used as a herbal medicine to treat colds, headaches, neuralgia, and arthralgia [66]. PMA/A23187-treated RPMCs were administered with 0.2, 0.5, and 1 mg/mL of OR extract for 30 min, while 0.5 and 1 mg/mL of the extract was administered to compound 48/80-treated RPMCs for 15 min. Downregulations in TNF-α, IL-6, p-ERK1/2, p-p38 MAPK, and p-IκBα were shown.

*Perilla frutescens* is a dietary leaf herb that has been used for Chinese medicine, known for its anti-inflammatory activity [67]. Kamei et al. reported that *Perilla*-derived methoxyflavanone prevented passive cutaneous anaphylaxis and allergic rhinitis-like nasal symptoms. *Perilla*-derived methoxyflavanone was treated to anti-DNP IgE-sensitized RBL-2H3 cells. Representative indicators such as p-Akt and intracellular Ca^2+^ influx were negatively regulated.

Effects and mechanism of 30% ethanol extract powder of *Perilla frutescens* var. acuta Kudo was observed [68]. It was treated to mouse mast cells and mouse eosinophils at a dose of 1 g/kg for 10 days. Additionally, PMACI-exposed HMC-1 was treated with 0.01, 0.1, and 1 mg/mL of EPPF for 1 h. The decoction initiated the degradation of IL-1β, IL-6, TNF-α, COX-2, and NF-κB.

Rosmarinic acid was treated to mouse mast cells and mouse eosinophils at a dose of 4 g/kg for 10 days, and 100 µM was treated to PMACI-exposed HMC-1 for 1 h [68]. The effects of rosmarinic acid were related to the degradation of IL-1β, IL-6, TNF-α IL-6, and NF-κB.

*Spinacia oleracea* Linn. extract was orally administered to Hydrogen peroxide-applied SH-SY5Y neuroblastoma cells at a dose of 25 μL/mL for 24 h [69]. Viable cell number recovery related to reduced levels of IL-4, IL-13, and Ig-E was observed.

Treatment of Ze339 at a dose of 3 µg/mg in human nasal epithelial cells from inferior turbinate of patients for 24 h inhibited the recruitment of inflammatory cells, pro-inflammatory cytokine, and chemokine response of viral mimics [70]. Ze339 increased the expression of granulocyte-colony stimulating factor (G-CSF) and decreased the expression of IL-8, chemokine (C-C motif) ligand 3 (CCL-3), IL-6, signal transducer, an activator of transcription 1 (STAT1), STAT3, and STAT6.

**Table 1:** In vitro studies—plant-originated natural products.

Compound/Extract	Source	Experimental Subject	Dose; Duration	Efficacy	Mechanism	Reference
*Albizia lebbeck* bark ethanol extract	*Albizia lebbeck*	PMA-treated HeLa	0.1, 1, 10 μg/mL; 24 h	Improvement of allergic nasal symptoms	↓ H1R, HDC	[55]
		Histamine-treated HeLa	10 μg/mL; 24 h			
Brazilian green propolis ethanol extract	*Baccharis dracunculifolia*	TDI-sensitized rats	40, 80 mg/kg; 3 weeks	Improvement of allergic nasal symptoms	↓ H1R, p-PKCδ, IL-9, IL-4, IL-5	[56]
		HeLa	25, 75, 100, 125, 200 µg/mL; 3 h		↓ H1R, p-PKCδ, IL-9	
		RBL-2H3	25, 75, 100 µg/mL; 3 h			
Brazilian green propolis ethanol extract	*Baccharis dracunculifolia*	Cry j1-treated PBMCs	3, 10, 30 μg/mL; 6 days	Prevention of the nasal obstruction	↓ IL-5, IL-13	[57]
		Cry j1-treated peripheral leukocytes from allergic human	3, 10, 30, 100 μg/mL; 6 days		↓ CysLTs	
Chrysin (5,7-dihydroxyflavone)	*Passiflora caerulea*	(Anti-DNP IgE)-sensitized RBL-2H3	0.1, 1, 10 µg/mL; 30 min	Inhibition of mast cell-derived allergic inflammatory reactions	↓ TNF-α, IL-1β, IL-4, IL-6, caspase-1, NF-κB	[58]
		PMA/A23187-treated RBL-2H3				
		PMA/A23187-treated HMC-1				
*Citrus limon* Burm. f.	*Citrus limon* (L.) Burm. f.	PBMCs from five grass pollen-allergic human	0.01 g/mL; 7 days	Reduction of allergic responses Attenuation of nasal inflammation	↑ IFN-γ ↓ TNF-α, IL-5	[60]
*Cydonia* oblonga Mill.	*Cydonia oblonga* Mill.	PBMCs from five grass pollen-allergic human	0.01 g/mL; 7 days	Reduction of allergic responses Attenuation of nasal inflammation	↑ IFN-γ	[60]
*Elsholtzia ciliata* (Thunb.) Hyl. (Labiatae) water extract	*Elsholtzia ciliata* (Thunb.) Hyl.	PMACI-exposed HMC-1	1, 10, 100 μg/mL; 30 min	Inhibition of mast cell-mediated allergic reactions Treatment of allergic inflammatory diseases	↑ IκBα ↓ TNF-α, IL-6, NF-κB, p-p38 MAPK	[61]
Enzyme-treated (protease-degraded) Royal Jelly	*Apis cerana* Fabricius	RBL-2H3	25, 75, 100 µg/mL; 3 h	Improvement of allergic nasal symptoms	↓ H1R, p-PKCδ, IL-9	[56]
		HeLa	25, 75, 100, 125, 200 µg/mL; 3 h			
Magnoliae Flos ethanol extract	Magnoliae Flos	hANO1-transfected HEK293T	0.1, 0.3, 1 mg/mL; 600 s	Prevention of allergic rhinitis		[62]
		HEK293T, Calu-3	30, 100, 300 μg/mL; 600 s			
Gencydo^®^	*Citrus limon* *, Cydonia oblonga*	(DNP–HSA)-treated RBL-2H3	0.2, 0.4, 0.8 mg/mL; 10 min	Inhibition of the release of histamine and degranulation	↓ IL-8, TNF-α	[63]
		(DNP–HSA)-treated mice BMMCs	0.2, 0.4, 0.8 mg/mL; 60 min			
		(TNF-α/IL-4)-treated human bronchial epithelial cells	0.2, 0.4, 0.8 mg/mL; 24 h	Inhibition of the release of eotaxin		
		PMA/A23187-activated human mast cells	0.2, 0.4, 0.8 mg/mL; 24 h	Orchestration of early- and late-phase allergic reaction		
		(GM-CSF)-treated human eosinophil granulocytes	0.2, 0.4, 0.8 mg/mL; 24 h	No impact on the behavior of (GM-CSF)-treated human eosinophils		
*Lindera obtusiloba* water extract	*Lindera obtusiloba*	PMACI-treated HMC-1	0.1, 1, 10, 100 μg/mL; 30 min	Reduction of histamine release Inhibition of systemic and local allergic reaction	↑ IκBα ↓ TNF-α, IL-6, NF-κB, histamine	[64]
		(DNP-HSA)-treated RBL-2H3				
		(DNP-HSA)-treated RPMCs				
Ortho-vanillic acid	*Amomum xanthioides*	RPMCs	1, 10, 100 nmol/L; 24 h	Suppression of the mast cell-mediated allergic inflammatory response	↓ TNF-α, IL-4, p-Lyn, p-Syk, p-Akt	[65]
		OVA-treated RBL-2H3	1, 10, 100 nmol/L; 24 h	Suppression of histamine release		
*Ostericum koreanum* root methanol extract		PMA/A23187-treated RPMCs	0.2, 0.5, 1 mg/mL; 30 min	Attenuation of disease progression as determined by nasal symptoms and histological changes of the nasal mucosa	↓ TNF-α, IL-6, p-ERK1/2, p-p38 MAPK, p-IκBα	[66]
		Compound 48/80-treated RPMCs	0.5, 1 mg/mL; 15 min	Decreased compound 48/80-induced release of histamine		
*Perilla*-derived methoxyflavanone	*Perilla frutescens*	(Anti-DNP IgE)-sensitized RBL-2H3	25–300 µM; 30 min	Inhibition of IgE-mediated histamine release	↓ p-Akt, intracellular Ca^2+^ influx	[67]
			100, 300 µM/2.5 mL; 30 min	Suppression of IgE-mediated mast cell degranulation		
*Perilla frutescens* var. acuta Kudo 30% ethanol extract powder	*Perilla frutescens* var.	Mouse mast cells	1 g/kg; 10 days	Amelioration of allergic inflammatory reactions	↓ IL-1β, IL-6, TNF-α, COX-2, NF-κB	[68]
		Mouse Eosinophils	1 g/kg; 10 days			
		PMACI-exposed HMC-1	0.01, 0.1, 1 mg/mL; 1 h			
Rosmarinic acid		Mouse mast cells	4 g/kg; 10 days	Amelioration of allergic inflammatory reactions	↓ IL-1β, IL-6, TNF-α, NF-κB	[68]
		Mouse Eosinophils	4 g/kg; 10 days			
		PMACI-exposed HMC-1	100 µM; 1 h			
*Spinacia oleracea* Linn. aqueous extract	*Spinacia oleracea* Linn.	(Hydrogen-peroxide)-applied SH-SY5Y neuroblastoma cells	25 μL/mL; 24 h	Viable cell number recovery	↓ IL-4, IL-13, Ig-E	[69]
Ze339	*Petasites hybridus*	Human nasal epithelial cells; inferior turbinates of patients	3 µg/mg; 24 h	Reduction of recruitment of inflammatory cells, Inhibition of pro-inflammatory cytokine and chemokine response to viral mimics	↑ G-CSF ↓ IL-8, CCL-3, IL-6, STAT1, STAT3, STAT6	[70]

TDI, toluene diisocyanate; PMA, Phorbol myristate acetate; IL, Interleukin; H1R, H1-receptor; HDC, histidine decarboxylase; AR, allergic rhinitis; IgE, Immunoglobulin E; OVA, Ovalbumin; Cry j1, crystal proteins j1; CysLTs, Cysteinyl leukotrienes; IFN-γ, Interferon-gamma; DNP, Dinitrophenyl; NF-κB, nuclear factor kappa-light-chain-enhancer of activated B cells; PBMC, peripheral blood mononuclear cells; IκBα, nuclear factor of kappa light polypeptide gene enhancer in B-cells inhibitor alpha; p-p38 MAPK, Phospho-p38 mitogen-activated protein kinases; COX, Cyclooxygenase; DNP-HSA, Dinitrophenyl-Human Serum Albumin; GM-CSF, Granulocyte-macrophage colony-stimulating factor; p-Syk, Phospho-Syk (Tyr525/526); p-Akt, Phospho-Akt (Ser473); p-ERK1/2, phospho-ERK1/2; STAT, Signal transducer and activator of transcription; G-CSF, Granulocyte-colony stimulating factor; CCL-3, Chemokine (c-C motif) ligand 3; ↑, up-regulation; ↓, down-regulation.

#### 3.1.2. In Vivo Studies

There were 25 in vivo studies of plant-originated natural products (Table 2). Nurul et al. orally administered 200 mg of powdered *Albizia lebbeck* bark ethanol extract to toluene-2, 4-diisocyanate (TDI)-sensitized allergy rats once a day for 3 weeks [55]. IL-4, IL-5, and IL-13 were decreased. *Albizia lebbeck* reduced the amount of sneezing and nasal rubbing, thus alleviating nasal symptoms.

*Artemisia vulgaris* water extract was orally administered in a mugwort pollen-sensitized AR BALB/c mouse model for 15 days [71]. Except for the control group, each group was treated with 10, 50, and 100 µg/mL of the extract, respectively. *Artemisia vulgaris* water extract significantly declined the level of IgE. It also controlled the imbalance between T helper cell 1 (Th1) and T helper cell 2 (Th2) cytokines by increasing IL-12 and decreasing IL-4 and IL-5. The extract attenuated the symptoms of allergic rhinitis in a dose-dependent manner.

Awa-tea inhibited allergic diseases as well as stimulating anti-obesity and anti-oxidant activity [72]. TDI-sensitized rats were treated with 40 mg/kg of Awa-tea leaf hot-water extract once a day for 3 weeks. Decreased levels of IL-9 and IL-4 were observed.

Berberine, originated from *Berberis*, has been widely used in China as a gastrointestinal remedy and is known for its antibacterial, antifungal, and anti-inflammatory properties [73,74,75,76]. *Dermatophagoides farinae* (Derf)-induced AR BALB/c mice were orally administered 10 µg/mL of berberine for 5 days [77]. Serum IgE, GATA3 mRNA levels, and tissue eosinophil counts decreased, while Foxp3 increased compared with the control group. Berberine showed anti-inflammatory activity regarding allergic rhinitis through regulating the mechanism and function of diverse cells.

Shaha et al. orally administered 40 and 80 mg/kg of Brazilian green propolis (BGPP) to TDI-sensitized rats once a day for 22 days [56]. BGPP treatment led to the degradation of H1R mRNA, p-PKCδ, IL-9, IL-4, and IL-5.

*Bupleurum chinense* water extract (BCE), derived from *Bupleurum chinense*, is found in East Asia and is commonly used as a traditional herbal medicine [78]. BCE was orally administered (50, 100, and 200 mg/kg for 11 days) in an ovalbumin (OVA)-induced AR BALB/c mouse model [79]. It increased the expression of Th1/regulatory T cells (Treg)-related cytokines such as IL-10, IL-12, and Interferon-gamma (IFN-γ). On the other hand, the secretion of Th2-related cytokines, IL-4, IL-5, and IL-13, including nasal lavage fluid eosinophil-specific chemoattractant Chemokine ligand 24 (CCL24) was suppressed.

Compound 40/80-induced systemic anaphylaxis mice and PCA mice were intraperitoneally administered with Chrysin (5, 7-dihydroxyflavone) at a dose range of 1–100 mg/kg 1 h before the challenge [58]. Chrysin blocked compound 40/80-induced fatal shock and reduced PCA reaction.

*Cinnamomum zeylanicum* bark hydroalcoholic extract has a long history of having been used as a traditional medicine to cure autoimmune diseases and cold in diverse regions such as India, China, Egypt, and Europe [80,81,82]. Aswar et al. induced AR in BALB/c mice by intranasal challenging OVA and injected 3, 10, and 30 µg/kg of *Cinnamomum zeylanicum* bark hydroalcoholic extract for 8 days [83]. The primary result was mast cell stabilization and subsequent prevention of IgE and histamine, which indicates that the extract effectively ameliorates nasal inflammation.

*Dryopteris crassirhizoma* (DC) is commonly used in Korea, Japan, and China to treat the common cold, tapeworm infection, and bacterial and inflammatory diseases [84]. OVA-induced AR BALB/c mice were treated with 100, 200, and 400 mg/kg of *Dryopteris crassirhizoma* ethanol extracts by oral gavage for 11 days [85]. DC increased the level of Treg cytokines in nasal fluid but decreased the activity of IgE, Immunoglobulin G1 (IgG1), and Immunoglobulin G2 (IgG2) cytokines in the blood. DC significantly prevented the occurrence of nasal allergic symptoms and the internal work of histamine.

TDI-sensitized rats were treated with 40 and 80 mg/kg of enzyme-treated (protease-degraded) royal jelly (3.5% (E)-10-hydroxy-2-decenoic acid and 0.6% 10-hydroxydecanoic acid) once a day for 22 days [56]. Decreased levels of H1R, p-PKCδ, IL-9, IL-4, and IL-5 were observed.

Epigallocatechin gallate, originated from *Camellia sinensis*, is a polyphenolic constituent of green tea catechins [86]. EGCG reduced the OVA-induced allergic rhinitis in female BALB/c mice at doses of 25, 50, and 100 mg/kg for 10 days. It inhibited the expression of IL-1β, IL-4, IL-6, and COX-2.

DNP-HSA-administered mouse bone marrow-derived mast cells (BMMCs) were treated with 0.2, 0.4, and 0.8 mg/mL of Gencydo^®^ for 60 min [63]. After treatment, IL-8 and TNF-α levels were decreased.

Various kinds of literature have already found that soybean suppressed the development of chronic diseases, as it contains cancer risk-reducing properties [87,88]. *Glycine max* water extract, obtained from dried green soybean powder, was administered in the TDI-induced guinea pig rhinitis model at the concentrations of 30, 150, and 300 µg/kg for 28 days [89]. Green soybean extracts decreased levels of IgE serum, IL-4, B-cell activating factor (BAFF), and a proliferation-inducing ligand (APRIL) production. It effectively acted as an immune modulator of allergic rhinitis, preventing nasal mucosa secretions and allergic symptoms.

*Lindera obtusiloba* water extract was intraperitoneally administered to compound 48/80-induced systemic allergy mice at doses of 1, 10, and 100 mg/kg one hour before the challenge [64]. The anti-allergic effects of the extract were related to reduced TNF-α, IL-6, IκBα, and NF-κB.

Ortho-vanillic acid was orally administered to OVA-induced active systemic anaphylaxis (ASA) mice at doses of 2, 10, and 50 mg/kg for 14 days [65]. Furthermore, IgE-mediated passive cutaneous anaphylaxis (PCA) mice were orally treated with 50 mg/kg of saline-dissolved ortho-vanillic acid for 1 h. Kim et al. concluded that ortho-vanillic acid attenuated the OVA-induced active systemic anaphylaxis and lessened IgE-mediated cutaneous allergic reactions.

Efficacy of Osterici Radix (OR) root methanol extract was demonstrated by orally administering OVA-induced allergic rhinitis BALB/c mice with doses of 50 or 100 mg/kg from day 28 to day 34 [66]. OR extract attenuated disease progression, which is determined by nasal symptoms and histological changes of the nasal mucosa. Degradation of OVA-specific IgE and Il-4 and the elevation of IFN-γ were related to the effects.

*Perilla frutescens* var. acuta Kudo 30% ethanol extract powder (EPPF) inhibited mast cell-mediated immediate-type allergic reactions [68]. OVA-sensitized allergic rhinitis mice were orally treated with 0.01, 0.1, and 1 g/kg of EPPF before intranasal OVA challenge for 10 days. IL-1β, IL-6, TNF-α, COX-2, and NF-κB levels were reduced.

*Piper nigrum* L. ethanol extract (PNE), originated from Piperine, is widely used for treating bacteria-induced illnesses, tumors, diabetes, and inflammation [90,91,92]. OVA-induced AR BALB/c mice were orally administered with 50, 100, and 200 mg/kg PNE or 2.5 mg/kg Dexamethasone (Dex) for 13 days, respectively [93]. *Piper nigrum* L. ethanol extract positively inhibited nasal symptoms by suppressing the accumulation of inflammatory cells. The activation of inflammation-related cytokines signal transducer and activator of transcription 3 (STAT3) and nuclear factor kappa-light-chain-enhancer of activated B cells protein 65 (NFκBp65) were inhibited, and the action of anti-inflammatory cytokine Th1 was elevated. PNE showed significant differences compared with the naïve group and had a similar effect when compared with the Dex group.

Another study was conducted to determine the effect of *Piper nigrum* L. in treating allergic rhinitis. OVA-induced AR Swiss albino mice were treated with either 10, 20, or 40 mg/kg of *Piper nigrum* L. extract or 10 mg/kg of montelukast for 7 days [94]. The extract reduced the expression of IL-6, IL-1β, and IgE in contrast with the control group.

Propolis is a transformed form of plant resin produced by honey bees and has been used to treat burns and wounds, gastric ulcers, and prostate hyperplasia [95,96]. It is widely known for its diverse anti-microbial, anti-viral, and anti-inflammatory functions [97,98,99,100]. Yasar et al. orally applied 200 mg/kg of propolis to a group of Sprague Dawley (SD) rats, while the other groups were given mometasone furoate, ketotifen, and saline (sham treatment) [101]. Propolis, mometasone furoate, and ketotifen similarly down-regulated ciliary loss, inflammation, number of goblet cells, vascular proliferation, eosinophil count, and allergic symptoms scores. In the long-term, the observation results of symptom scores implied that propolis had a more powerful efficacy of inhibiting allergic reactions than sham medicines.

Rosae Multiflorae Fructus water extract (RMFE), originated from Rosae Multiflorae Fructus, is known for its anti-oxidative, analgesic, and anti-inflammatory properties [102]. Oral administration of 100, 200, and 400 mg/kg RMFE was implemented in an OVA-induced AR BALB/c mouse model for 13 days [103]. RMFE increased the Th1-associated cytokine IL-12 while inhibiting the action of Th2 related cytokines IL-4, IL-5, and IL-13 in nasal lavage fluid. Additionally, components of RMFE including hyperoside, isoquercitrin, and miquelianin were found to prevent IL-4 secretion.

OVA-sensitized allergic rhinitis mice were orally administered with 4 mg/kg of Rosmarinic acid for 10 days, which led to the degradation of IL-1β, IL-6, TNF-α IL-6, and NF-κB [68].

*Spinacia oleracea* Linn. aqueous extract, which is well-known for its vitamin content, improved the asthmatic symptoms induced by the OVA challenge and reduced the BAL’s eosinophil expression [69]. OVA-challenge asthmatic mice were orally treated with *Spinacia oleracea* Linn. extract at a dose of 25 μL/mL for 24 h.

*Syzygium formosum* (Wall.) Masam. leaves have been routinely used among indigenous Vietnamese people to treat various allergic symptoms, including dermatitis and rhinitis [104]. They also improved food allergy symptoms and inflammatory lesions in the gut. *Syzygium formosum* leaves extract was orally administered to chicken ovalbumin (cOVA)-induced food allergy mice at doses of 80 and 200 mg/kg for 13 days. T helper 2 cell cytokines (Th2 cytokines) (e.g., IL-4, IL-5, IL-10), and multiple c2 domains and transmembrane region proteins-1 (MCTP-1) were significantly decreased.

Xanthii Fructus, the ripe fruits of *Xanthium sibiricum* Patr., is a well-known herb used as traditional Chinese medicine [105]. OVA-sensitized Male BALB/C mice were orally treated with 1 g/kg of stir-fried Xanthii Fructus ethanol extract for 15 days. Phosphatidylcholine 40:8 (PC (40:8)) and L-valine were increased, thereby soothing nasal orifices and eliminating wind-dampness.

Wild grape (*Ampelopsis glandulosa*) has been reported to have anti-inflammatory, anti-hepatotoxic, and anti-osteoclastogenesis activity [72]. Islam et al. demonstrated that wild grape hot-water extract alleviated nasal symptoms and eosinophilic inflammation. The extract was orally treated to TDI-sensitized rats at doses of 25 and 50 mg/kg once a day, for 3 weeks. It inhibited the expression of p-PKCδ, H_1_R, IL-9, IL-4, IL-5, and IL-33.

**Table 2:** In vivo studies—plant-originated natural products.

Compound/Extract	Source	Experimental Subject	Dose; Duration	Efficacy	Mechanism	Reference
*Albizia lebbeck* bark ethanol extract	*Albizia lebbeck*	TDI-sensitized allergy rats	200 mg; 3 weeks	Reduction of the amount of sneezing and nasal rubbing.	↓ IL-4, IL-5, IL-13	[55]
*Artemisia vulgaris* water extract	*Artemisia vulgaris*	Mugwort pollen-injected AR BALB/c mice	10, 50, 100 µg/mL; 15 days	Reduction of allergic inflammation	↑ IL-12 ↓ IL-4, IL-5, IgE2, IgE	[71]
Awa-tea leaves hot water extract	*Setaria italica*	TDI-sensitized rats	40 mg/kg; 21 days	Inhibition of the allergic diseases	↓ IL-9, IL-4	[72]
Berberine	*Berberis*	(*Dermatophagoides farinae* (Derf)-OVA)-induced AR BALB/c mice	10 µg/mL; 57 days	Reduction of allergic inflammation	↑ Foxp3	[77]
Brazilian green propolis ethanol extract	*Baccharis dracunculifolia*	TDI-sensitized rats	40, 80 mg/kg; 3 weeks	Improvement of allergic nasal symptoms	↓ H1R, p-PKCδ, IL-9, IL-4, IL-5	[56]
*Bupleurum chinense* water extract	*Bupleurum chinense*	OVA-induced AR BALB/c mice	50, 100, 200 mg/kg; 11 days	Attenuation of allergic responses Suppression of nasal inflammation	↑ IL-10, IL-12, IFN-γ ↓ IL-4, IL-5, IL-13, CCL24	[79]
Chrysin (5,7-dihydroxyflavone)	*Passiflora caerulea*	Compound 48/80-induced systemic anaphylaxis mice	1–100 mg/kg; 1 h	Blockage of compound 48/80-induced fatal shock		[58]
		PCA mice		Reduction of PCA reaction		
*Cinnamomum zeylanicum* bark hydroalcoholic extract	*Cinnamomum zeylanicum*	OVA-induced AR BALB/c mice	3, 10, 30 µg/kg; 8 days	Attenuation of nasal inflammation	↓ IgE, histamine	[83]
*Dryopteris crassirhizoma* ethanol extract	*Dryopteris crassirhizoma*	OVA-induced AR BALB/c mice	100, 200, 400 mg/kg; 11 days	Amelioration of nasal inflammation	↓ IgE, IgG1, IgG2	[85]
Enzyme-treated (protease-degraded) Royal Jelly	*Apis cerana* Fabricius	TDI-sensitized rats	40, 80 mg/kg; 3 weeks	Improvement of allergic nasal symptoms	↓ H1R, p-PKCδ, IL-9, IL-4, IL-5	[56]
Epigallocatechin gallate	*Camellia sinensis*	OVA-induced mice	25, 50, 100 mg/kg; 10 days	Reduction of allergic rhinitis	↓ IL-1β, IL-4, IL-6, COX-2	[86]
*Glycine max* water extract	*Glycine max*	(2,4-TDI)-induced rhinitis guinea pigs	30, 150, 300 µg/kg; 28 days	Suppression of nasal inflammation Decrease of allergic responses	↓ IgE	[89]
*Lindera obtusiloba* water extract	*Lindera obtusiloba*	Compound 48/80-induced systemic allergy mice	1, 10, 100 mg/kg; 1 h	Reduction of histamine release Inhibition of systemic and local allergic reaction	↑ IκBα ↓ TNF-α, IL-6, NF-κB	[64]
Ortho-vanillic acid	*Amomum xanthioides*	OVA-induced ASA mice	2, 10, 50 mg/kg; 14 days	Attenuation of the ovalbumin-induced ASA	↓ TNF-α, IL-4, p-Lyn, p-Syk, p-Akt	[65]
		IgE-mediated PCA mice	50 mg/kg; 1 h	Attenuation of the IgE mediated cutaneous allergic reactions		
*Ostericum koreanum* root methanol extract	*Ostericum koreanum*	OVA-induced allergic rhinitis BALB/c mice	50, 100 mg/kg; 7 days	Attenuation of disease progression as determined by nasal symptoms and histological changes of the nasal mucosa	↑ IFN-γ ↓ OVA-specific IgE, IL-4	[66]
*Perilla frutescens* var. acuta Kudo 30% ethanol extract powder	*Perilla frutescens* var.	OVA-sensitized allergic rhinitis mice	0.01, 0.1, 1 g/kg; 10 days	Amelioration of allergic inflammatory reactions	↓ IL-1β, IL-6, TNF-α, COX-2, NF-κB	[68]
*Piper nigrum* L. ethanol extract	*Piper nigrum* L.	OVA-induced AR BALB/c mice	50, 100, 200 mg/kg; 13 days	Inhibition of allergic nasal symptoms Suppression of nasal inflammation	↓ STAT3, NFκBp65	[93]
*Piper nigrum* L. extract	*Piper nigrum* L	OVA-induced AR Swiss albino mice	10, 20, 40 mg/kg; 7 days	Exhibits immunomodulatory and anti-inflammatory activity	↓ IL-6, IL-1β, IgE	[94]
Propolis		SD rats	200 mg/kg; 21 days	Inhibition of allergic symptoms		[101]
Rosae Multiflorae Fructus water extract	Rosae Multiflorae Fructus	OVA-induced AR BALB/c mice	100, 200, 400 mg/kg; 13 days	Inhibition of allergic symptoms Immunomodulatory effect on allergic rhinitis	↑ IL-12 ↓ IL-4, IL-5, IL-13	[103]
Rosmarinic acid		OVA-sensitized allergic rhinitis mice	4 mg/kg; 10 days	Amelioration of allergic inflammatory reactions	↓ IL-1β, IL-6, TNF-α, NF-κB	[68]
*Spinacia oleracea* Linn. aqueous extract	*Spinacia oleracea* Linn.	OVA-challenge asthmatic mice	25 μL/mL; 24 h	Improvement of the asthmatic symptoms induced by OVA-challenge Reduction of the BAL’s eosinophil expression	↓ IL-4, IL-13, Ig-E	[69]
*Syzygium formosum* leaves extract	*Syzygium formosum* (Wall.) Masam.	cOVA-induced food allergy mice	80, 200 mg/kg; 13 days	Improvement of the food allergy symptoms and the inflammatory lesion in the gut.	↓Th2 cytokines (e.g., IL-4, IL-5, IL-10), MCTP-1	[104]
Xanthii Fructus ethanol extract	*Xanthium sibiricum* Patr.	OVA-sensitized Male BALB/C mice	1 g/kg; 15 days	Alleviation of nasal orifices and elimination of wind-dampness	↑ PC(40:8), L-valine	[105]
Wild grape hot water extract	*Ampelopsis glandulosa*	TDI-sensitized rats	25, 50 mg/kg; 21 days	Alleviation of the nasal symptoms Alleviation of the eosinophilic inflammation	↓ p-PKCδ, H1R, IL-9, IL-4, IL-5, IL-33	[72]

TDI, toluene diisocyanate; IL, Interleukin; H1R, H1-receptor; AR, allergic rhinitis; IgE, Immunoglobulin E; Derf, Dermatophagoides farinae; OVA, Ovalbumin; Foxp3, forkhead box P3; IFN-γ, Interferon-gamma; CCL24, Chemokine ligand 24; PCA, Passive cutaneous anaphylaxis; NF-κB, nuclear factor kappa-light-chain-enhancer of activated B cells; IgG1, Immunoglobulin G1; IgG2, Immunoglobulin G2; IκBα, nuclear factor of kappa light polypeptide gene enhancer in B-cells inhibitor alpha; COX, Cyclooxygenase; ASA, active systemic anaphylaxis; p-Syk, Phospho-Syk (Tyr525/526); p-Akt, Phospho-Akt (Ser473); STAT, signal transducer and activator of transcription; NFκBp65, nuclear factor kappa-light-chain-enhancer of activated B cells protein 65; PC(40:8), phosphatidylcholine 40:8; Th2, T helper 2 cell; MCTP-1, multiple c2 domains and transmembrane region proteins-1; ↑, up-regulation; ↓, down-regulation.

### 3.2. Fungi and Minerals-Originated Natural Products

Although plant-derived natural products are the most abundant materials for AR treatments, several studies cover compounds derived from fungi and minerals.

#### 3.2.1. In Vitro Studies

There were two in vitro studies of fungi and minerals (Table 3). Amber has been used to improve muscle pain, headaches, skin allergy, etc. [106]. RBL-2H3 cells were treated with 2 μL of 25 μg/mL Kuji amber methanol extract for 30 min. The extract induced the degradation of phospho-ERK1/2 (p-ERK1/2) and LTC_4_, thus inhibiting nasal blockage degranulation of RBL-2H3 cells. Meanwhile, kujigamberol (15,20-dinor-5,7,9-labdatrien-18-ol), isolated from Kuji amber, was also treated to RBL-2H3 cells in the same way as the Kuji amber methanol extract.

#### 3.2.2. In Vivo Studies

There were three in vivo studies of fungi and minerals (Table 4). *Ganoderma lucidum* has long been used in East Asia as a remedy for cancer, hypertension, hepatitis, hyperglycemia, and bronchitis [107,108,109]. Cedar pollen extract-sensitized AR guinea pig models were orally administered with *Ganoderma lucidum* powder suspended in water at the doses of 100 and 1000 mg/5 mL/kg for 8 weeks [110]. Treatment of *Ganoderma lucidum* effectively inhibited nasal inflammatory symptoms via suppressing CysLT1 receptor activation.

OVA-induced rhinitis guinea pigs were intranasally administered 20 μg of Kuji amber methanol extract for 10 min, 1 h after OVA exposure [106]. Additionally, Kujigamberol (15,20-dinor-5,7,9-labdatrien-18-ol) was orally administered to OVA-induced rhinitis guinea pigs. Both experiments resulted in the degradation of p-ERK1/2 and LTC_4_.

### 3.3. Decoctions of Natural Products

A decoction is an extract obtained by boiling one or more herbal materials for medical purposes. Various studies reported that decoctions have anti-AR effects in several experimental models.

#### 3.3.1. In Vitro Studies

There were four in vitro studies of decoctions (Table 5). Gami-hyunggyeyeongyotang is a traditional decoction used for treating allergic disorders [111]. Human mast cell line (HMC-1) cells were treated with Gami-hyunggaeyeongyotang at doses of 1, 10, or 100 μg/mL for an hour. Gami-hyunggaeyeongyotang alleviated the nasal symptoms of AR, such as sneezing, by suppressing IgE, IL-5, IL-6, IL-1β, monocyte chemoattractant protein (MCP)-1, macrophage inflammatory protein (MIP)-2, and caspase-1.

Mahuang Fuzi Xixin had an anti-inflammatory effect by decreasing PGE_2_ [112]. IgE-activated rat basophilic leukemia (RBL-2H3) cells were treated with M136 at doses of 3, 10, or 30 μM, M188 at doses of 5 or 10 μM, M234 at doses of 2 or 5 μM, and M289 at doses of 3, 10, or 30 μM for 1 h.

The expression of Toll-like receptor 4 (TLR4) and production of IL-12 increased when RAW264.7 was pre-stimulated by Senn-kinn-naidaku-sann, thus inhibiting the inflammatory effects [113]. RAW264.7 was treated at doses of 0.01, 0.1, 1, and 10 μg/mL for 24 h.

Shenqi affected RBL-2H3 cells, decreasing β-hexosaminidase and histamine when treated at doses of 50, 100, and 200 μg/mL for 1 h [114].

#### 3.3.2. In Vivo Studies

There were seventeen in vivo studies of decoctions (Table 6). Biyeom-Tang has been used to treat allergic rhinitis (AR) traditionally [115]. The ethanol extract of Biyeom-Tang inhibited allergic and inflammatory responses. The decoction suppressed β-hexosaminidase (β-Hex) when treated to BMMC from BALB/c mice at doses of 12.5, 25, or 50 μg/mL for an hour. Ethanol extract of Biyeom-Tang also suppressed the production of prostaglandin D_2_ (PGD_2_) and LTC_4_ on BMMC in BALB/c mice when the mice were treated with 6.3, 12.5, or 25 μg/mL of the extract for one hour. Additionally, the extract was orally administered with doses of 50, 100, or 200 mg/kg to OVA-induced AR BALB/c mice for 7 days. It was found that levels of IL-4, IL-5, IL-10, and IL-13 decreased.

Biyuanling Granules were orally administered to TDI-induced AR guinea pigs at doses of 4, 8, or 16 mg/g for 7 days once a day [116]. The decoction inhibited the inflammatory response by increasing Bcl-2 Associated X Protein (Bax) expression and suppressing IL-4, human SARS-specific immunoglobulin (SIgE), tumor necrosis factor α (TNF-α), pulmonary surfactant associated protein (SP), vascular cell adhesion molecule-1 (VCAM-1), and transcription factor p65 (P65).

Bu-Zhong-Yi-Qi-Tang improved the AR-like symptoms by increasing interferon-gamma (IFN-γ), Foxp3 and decreasing IgE, IL-4, and cluster of differentiation 4 (CD4) in OVA- and alum-induced AR BALB/c mice [117]. Group I mice were gavage-fed 10 g/100 mL of Bu-Zhong-Yi-Qi-Tang for 10 days, and Group II for 19 days.

OVA-induced AR BALB/c mice were orally administered 134 mg/kg of Gami-hyunggyeyeongyotang for 11 days [111]. IgE, IL-5, IL-6, IL-1β, MCP-1, MIP-2, and caspase-1 decreased, improving the symptoms of AR.

Hyeonggaeyeongyo-Tang is a traditional treatment for otolaryngology symptoms [118]. The decoction suppressed the progression of AR by decreasing IL-4, IL-13, and leukemia inhibitory factor (LIF). OVA-induced AR BALB/c mice were orally administered Hyeonggaeyeongyo-Tang at doses of 101, 202, or 404 mg/kg for 14 days.

KOB03 has been commonly used to treat hyperhidrosis, allergic asthma, and allergic rhinitis [119,120,121]. OVA-induced AR BALB/c mice and SD rats were orally administered 100 or 200 mg/kg of KOB03 for 7 days. The secretion of cytokines TNF-α, IL-1β, IL-6, and IL-8 were reduced [122]. These results confirmed that the decoction prohibits IgE production and Th2 cytokines, thus regulating symptoms of allergic inflammations.

In another study, 500 mg/kg of KOB03 was orally injected into OVA-induced AR BALB/c mice for 8 consecutive days [123]. KOB03 controlled the levels of IgE, LTC_4_, IL-4, and IL-1β, thus significantly increasing Th1 cytokine and down-regulating Th2 cytokine. KOB03 showed its efficacy in inhibiting nasal inflammation by controlling diverse allergic mediators and Th1/Th2 balance.

Mahuang Fuzi Xixin decoction alleviated the symptoms of AR in OVA-induced AR specific pathogen-free (SPF) Wistar rats when orally administered in quantities of 1.52 g/mL daily for 10 days [124]. Mahuang Fuzi Xixin decoction reduced IgE, histamine, IFN-γ, IL-4, GATA3, and STAT6.

OVA-induced AR BALB/c mice were orally administered 1 mg/mL of Pyeongwee-San for 10 days [125]. The decoction increased IFN-γ and decreased IgE, IL-4, TNF-α, IL-1β, MIP-2, intercellular adhesion molecule 1 (ICAM-1), and COX-2.

The effect of Qi-fang-bi-min-tang combined with mesenchymal stem cells (MSCs) on AR treatment was investigated [126]. OVA-induced AR SD rats were injected with 5 × 10^6^ MSCs and 2% of qi-fang-bi-min-tang for 19 days, resulting in the inhibition of AR symptoms by suppressing IFN-γ, IL-17, IL-4, TNF-α, and IgE. The equal amount of MSCs and Qi-fang-bi-min-fang showed a similar effect when tested in OVA-induced AR BALB/C mice. Additionally, TNF-α, IL-6, IFN-γ, IL-4, and IL-17 were suppressed by 20% of Qi-fang-bi-min-tang.

Senn-kinn-naidaku-sann alleviated the symptoms of AR in OVA-induced AR C3H/HeN mice by increasing IFN-γ and decreasing IgE, immunoglobulin G1 (IgG1), IL-4, and IL-5 [113]. The mice were orally administered with 100 and 1000 mg/kg of the decoction for 7 days. The same dose had no enhancement of AR in OVA-induced AR C3H/HeJ mice.

OVA-sensitized primary spleen lymphocytes of Wistar rats were treated with 50, 100, and 200 μg/mL of Shenqi for 7 days [114]. Increased T-bet and decreased GATA3, p-STAT6, and suppressor of cytokine signaling 1 (SOCS1) were observed. Meanwhile, Shenqi on OVA-induced AR Wistar rats at doses of 0.9, 2.7, and 8.1 g/kg for 7 days increased IFN-γ, T-bet, and suppressed IL-4, IgE, GATA3, phospho-STAT6 (p-STAT6), and SOCS1.

OVA-induced AR BALB/c mice were fed with 20 mg/mL of So-Cheong-Ryong-Tang for 14 days [127]. So-Cheong-Ryong-Tang suppressed the progression of AR by decreasing IL-4, IL-6, IL-1β, TNF-α, and LIF. In another study, OVA-and-alum-induced AR BALB/c mice were orally administered with 1 g/kg of So-Cheong-Ryong-Tang per day for 7 days [128]. So-Cheong-Ryong-Tang alleviated the symptoms of AR such as nasal rubbing and sneezing by increasing levels of IgG2a and IFN-γ while decreasing levels of IgE, IL-4, IgG1, and IL-5.

Tong Qiao is new Chinese medicine developed by Chengdu Huashen Zhiyao, Ltd. Co., [129]. OVA-induced AR SD rats were given intranasal drops of Tong Qiao, 10 µL/kg per nostril per treatment, three times a day, for 7 or 15 days. Tong Qiao alleviated the symptoms of AR by suppressing eotaxin, IL-5, and IL-13.

Xingbi gel is a traditional application prescribed for the cure of allergic rhinitis [130]. OVA-induced AR guinea pig models were sensitized with 50 µL/kg of Xingbi gel for 12 days. Xingbi gel regulated the secretion of Leukotriene E4 (LTE_4_) and IgE, which led to the decreased production of inflammatory mediators and cytokines.

Yiqi Wenyang Fang is known to treat AR patients with a cold deficiency in the lung and spleen [131]. Yiqi Wenyang Fang inhibited inflammatory response and alleviated the symptoms of AR in OVA-induced AR SD rats by decreasing IL-10, transforming growth factor-beta 1 (TGF-β1), IL-4, and IL-13. The rats were given 1.6 g/mL of Yiqi Wenyang Fang per day, by gavage, for 28 days.

**Table 6:** In vivo studies—decoctions of natural products.

Name	Constitutions	Experimental Subject	Dose; Duration	Efficacy	Mechanism	Reference
Biyeom-Tang (ethanol extract)	Xanthii Fructus (*Xanthium strumarium L.)*, Trichosanthis Semen (*Trichosanthes kirilowii* Maxim.), Angelicae Dahuricae Radix, Menthae Herba (*Mentha arvensis* L.)	BMMCs from BALB/c mice	12.5, 25, 50 μg/mL; 1 h	Inhibition of allergic and inflammatory effects	↓ β-Hex	[115]
		BMMCs from BALB/c mice	6.3, 12.5, 25 μg/mL; 1 h		↓ PGD_2_, LTC_4_	
		OVA-induced AR BALB/c mice	50, 100, 200 mg/kg; 7 days		↓ IL-4, IL-5, IL-10, IL-13	
Biyuanling	*Xanthium strumarium* subsp. *sibiricum* (Patrin ex Widder) Greuter, *Lonicera japonica* Thunb., *Glycyrrhiza uralensis* Fisch., *Scutellaria baicalensis* Georgi	TDI-induced AR Guinea pigs	4, 8, 16 mg/g; 7 days	Inhibition of inflammatory response	↑ Bax ↓ IL-4, SIgE, TNF-α, SP, VCAM-1, P65	[116]
Bu-Zhong-Yi-Qi-Tang	Astragali Radix, Ginseng Radix, *Atractylodes macrocephala* Koidz., Glycyrrhizae Radix, Cimicifugae Rhizoma, Bupleuri Radix, Aurantii Nobilis Pericarpium Citri Unshius Pericarpium, finger citron, Chinese magnolia vine fruit	(OVA-and-alum)-induced AR BALB/c mice	10 g/100 mL; 10, 19 days	Improvement of the AR-like symptoms	↑ IFN-γ, Foxp3 ↓ IgE, IL-4, CD4	[117]
Gami-hyunggyeyeongyotang	Schizonepetae Spica (*Nepeta tenuifolia* Benth.), Forsythiae Fructus (*Forsythia viridissima* Lindl.), Saposhnikoviae Radix (*Saposhnikovia divaricata* (Turcz.) Schischk.), Angelicae Radix (*Angelica acutiloba* Kitagawa), Cnidii Rhizoma (*Cnidium officinale* Makino), Paeoniae Radix Alba (*Paeonia lactiflora* Pallas), Angelicae Dahuricae Radix (*Angelica dahurica Bentham* et Hooker), Bupleuri Radix (*Bupleurum falcatum* Linne), Scutellariae Radix (*Scutellaria baicalensis* Georgi), Aurantii Fructus Immaturus (*Citrus* *aurantium* Linne), Gardeniae Frutus (*Gardenia jasminoides* Ellis), Platycodi Radix (*Platycodon grandiflorum* A. De candole), Glycyrrhizae Radix (*Glycyrrhiza uralensis* Fischer), Ulmi Cortex (*Ulmus macrocarpa* Hance), Xanthii Fructus (*Xanthium strumarium* Linne), Magnoliae Flos (*Magnolia kobus* De Candolle), Rubiae Radix (*Rubia akane* Nakai)	OVA-induced AR BALB/c mice	134 mg/kg; 11 days	Alleviation of the symptoms of AR	↓ IgE, IL-5, IL-6, IL-1β, MCP-1, MIP-2, caspase-1	[111]
Hyeonggaeyeongyo-Tang	Schizonepeta Spica, Forsythiae Fructus, Saposhnikoviae Radix, Angelicae Gigantis Radix, Cnidii Rhizoma, Paeoniae Radix Alba, Bupleuri Radix, Aurantii Fructus, Scutellariae Radix, Gardeniae Fructus, Angelicae Dahuricae Radix, Platycodi Radix, Glycyrrhizae Radix	OVA-induced AR BALB/c mice	101, 202, 404 mg/kg; 14 days	Suppression of the progression of AR	↓ IL-4, IL-13, LIF	[118]
KOB03	Atractylodis Rhizoma Alba (*Atractylodes japonica* Koidz)*,* Astragalus Radix (*Astragalus membranaceus* Bunge), Saposhnikoviae Radix (*Saposhnikovia divaricata* Schischkin), Osterici Radix (*Ostericum koreanum* maximowicz), Scutellariae Radix (*Scutellaria baicalensis* Georgi)	OVA-induced AR BALB/c mice	100, 200 mg/kg; 7 days	Regulation of allergic inflammation	↓ TNF-α, IL-1β, IL-6, IL-8	[122]
		OVA-induced AR SD rats				
KOB03	Atractylodis Rhizoma White (*Atractylodes japonica* Koidz)*,* Astragalus Root (*Astragalus membranaceus* Bunge), Saposhnikovia Root (*Saposhnikovia divaricata* Schischkin), Ostericum Root (*Ostericum koreanum* maximowicz), Scutellaria Root (*Scutellaria baicalensis* Georgi)	OVA-induced AR BALB/c mouse model	500 mg/kg; 8 days	Inhibition of nasal inflammation	↓ IgE, LTC_4_, IL-4, and IL-1β	[123]
Mahuang Fuzi Xixin Decoction	Ephedrae, Aconiti Lateralis Radix, Asarum	OVA-induced AR SPF Wistar rats	1.52 g/mL; 10 days	Alleviation of the symptoms of AR	↓ IgE, histamine, IFN-γ, IL-4, GATA3, STAT6	[124]
Pyeongwee-San extract (KMP6)	*Atractylodes japonica* Koidz. ex Kitam., *Machilus thunbergii* Siebold & Zucc., *Citrus reticulata* Blanco, *Zizyphus jujube, Glycyrrhiza uralensis* Fisch., *Zingiber officinale* Roscoe	OVA-induced AR BALB/c mice	1 mg/mL; 10 days	Amelioration of AR	↑ IFN-γ ↓ IgE, IL-4, TNF-α, IL-1β, MIP-2, ICAM-1, COX-2	[125]
Qi-fang-bi-min-tang (QFBMT)	Astragali Radix, Ramulus Cinnamomi, Saposhnikoviae Radix, Magnoliae Flos, Atractylodis Macrocephalae Rhizoma, Zingiberis Rhizoma, Paeoniae Radix Alba, Glycyrrhizae Radix	OVA-induced AR SD rats	5 × 10^6^ MSCs, 2% QFBMT; 19 days	Inhibition of AR symptoms combined with MSCs	↓ IFN-γ, IL-17, IL-4, TNF-α, IgE	[126]
		OVA-induced AR BALB/c mice	QFBMT + MSCs: 5 × 10^6^ MSCs, 2%	Inhibition of AR symptoms combined with MSCs	↓ TNF-α, IL-6, IFN-γ, IL-4, IL-17	
			QFBMT: 20%; 19 days	Inhibition of AR		
Senn-kinn-naidaku-sann	Ginseng Radix, Angelicae Radix, Astragali Radix, Cnidii Rhizoma, Sinomeni Caulis et Rhizoma, Platycodonis Radix, Magnoliae Cortex, Angelicae Dahuricae Radix, Glycyrrhizae Radix	OVA-induced AR C3H/HeN mice	100, 1000 mg/kg; 7 days	Alleviation of the symptoms of AR	↑ IFN-γ	[113]
		OVA-induced AR C3H/HeJ mice	100, 1000 mg/kg; 7 days	No enhancement of AR	↓ IgE, IgG1, IL-4, IL-5	
Shenqi	Angelicae Dahuricae Radix (*Angelica dahurica* (Hoffm.) Benth. & Hook.f. ex Franch. & Sav.)*,* Scutellariae Radix (*Scutellaria baicalensis* Georgi)*,* Lonicerae Japonicae Flos (*Lonicera japonica* Thunb.)*,* Menthae Haplocalycis Herba (*Mentha canadensis* L.), Astragali Radix (*Astragalus propinquus* Schischkin)*,* Codonopsis Radix (*Codonopsis pilosula* subsp. *tangshen* (Oliv.) D.Y.Hong)*,* Isatidis Folium (*Isatis tinctoria* L.)*,* Taraxaci Herba (*Taraxacum mongolicum* Hand.-Mazz.)*,* Agrimoniae Herba (*Agrimonia pilosa* Ledeb.), Asiasari Radix et Rhizome (*Asarum sieboldii* Miq.)	OVA-sensitized primary spleen lymphocytes of Wistar rats	50, 100, 200 μg/mL; 7 days	Alleviation of symptoms of AR	↑ T-bet ↓ GATA3, p-STAT6, SOCS1	[114]
		OVA-induced AR Wistar rats	0.9, 2.7, 8.1 g/kg; 7 days		↑ IFN-γ, T-bet ↓ IL-4, IgE, GATA3, p-STAT6, SOCS1	
So-Cheong-Ryong-Tang	*Asarum sieboldii* Miq., *Glycyrrhiza uralensis* Fisch., *Ephedra sinica* Stapf, *Cinnamomum cassia* Blume, Peony root, *Pinellia ternata* (Thunb.) Makino, *Schisandra chinensis* (Turcz.) Baill., *Zingiber officinale* Roscoe	OVA-induced AR BALB/c mice	20 mg/mL; 14 days	Suppression of the progression of AR	↓ IL-4, IL-6, IL-1β, TNF-α, LIF	[127]
So-Cheong-Ryong-Tang	Pinelliae Tuber (*Pinellia ternata* (Thunb.) Makino), Ephedrae Herba (*Ephedra sinica* Stapf), Schisandrae Fructus (*Schisandra chinensis* (Turcz.) Baill.), Paeoniae Radix (*Paeonia lactiflora* Pall.), Glycyrrhizae Radix (*Glycyrrhiza uralensis* Fisch.), Zingiberis Siccatum Rhizoma (*Zingiber officinale* Roscoe), Asiasari Radix (*Asarum sieboldii* Miq.), Cinnamomi Cortex (*Cinnamomum cassia* (L.) J.Presl)	(OVA-and-alum)-induced AR BALB/c mice	1 g/kg; 7 days	Alleviation of the symptoms of AR	↑ IgG2a, IFN-γ ↓ IgE, IL-4, IgG1, IL-5	[128]
Tong Qiao	Angelicae Dahuricae Radix, *Gleditsia sinensis* Lam., Magnoliae Flos	OVA-induced AR SD rats	10 µL/kg; 7,15 days	Alleviation of the symptoms of AR	↓ eotaxin, IL-5, IL-13	[129]
Xingbi gel	Cynanchi Paniculati Radix (*Cynanchum paniculatum* (Bunge) Kitag. ex H.Hara), Cicadidae Periostracum (cicada slough), Bovis Calculus (bezoar), Borneolum Syntheticum (borneol)	OVA-induced AR guinea pigs	50 µL/kg; 12 days	Alleviation of the symptoms of AR	↓ LTE_4_, IgE	[130]
Yiqi Wenyang Fang	*Astragalus membranaceus* (Fisch.) Bunge, *Codonopsis pilosula* (Franch.) Nannf., Zingiberis Rhizoma, Cinnamomi Ramulus, *Ephedra sinica* Stapf, *Schisandra chinensis* (Turcz.) Baill.	OVA-induced AR SD rats	1.6 g/mL; 28 days	Inhibition of inflammatory response Alleviation of the symptoms of AR	↓ IL-10, TGF-β1, IL-4, IL-13	[131]

BMMC, bone-marrow derived mast cell; OVA, ovalbumin; AR, allergic rhinitis; β-Hex, β-hexosaminidase; PGD2, prostaglandin D2; LTC_4_, leukotriene C4; IL-4, interleukin 4; IL-5, interleukin 5; IL-10, interleukin 10; IL-13, interleukin 13; TDI, Toluene-2, 4-diisocyanate; Bax, Bcl-2 Associated X Protein; SIgE, human SARS-specific immunoglobulin; TNF-α, tumor necrosis factor α; SP, pulmonary surfactant associated protein; VCAM-1, vascular cell adhesion molecule-1; P65, transcription factor p65; IFN-γ, interferon-gamma; Foxp3, forkhead box P3; IgE, immunoglobulin E; CD4, cluster of differentiation 4; IL-6, interleukin 6; IL-1β, interleukin 1 beta; MCP-1, monocyte chemoattractant protein; MIP-2, macrophage inflammatory protein; LIF, leukemia inhibitory factor; SPF, specific pathogen-free; GATA3, GATA binding protein 3; STAT6, signal transducer and activator of transcription 6; ICAM-1, intercellular adhesion molecule 1; COX-2, cyclooxygenase 2; SD, Sprague Dawley; MSC, mesenchymal stem cell; IgG1, immunoglobulin G1; T-bet, T-box-expressed-in-T-cells; p-STAT6, phospho-STAT6; SOCS1, suppressor of cytokine signaling 1; IgG2a, immunoglobulin G2a; LTE_4_, Leukotriene E4; ↑, up-regulation; ↓, down-regulation.

### 3.4. Clinical Trials

Several clinical trials with natural products from plants and animals were conducted (Table 7). Depigoid 50% Grasses/50% Olea europaea or Depigoid 50% Grasses/50% *Parietaria judaica* were administered to patients with allergic rhinitis or rhinoconjunctivitis with or without seasonal asthma [132]. This trial was a phase II, prospective, open uncontrolled, and non-randomized study. The study evaluated the safety and tolerance of a rush build-up of administration of Depigoid forte pollen with the first maintenance dose administered 4 weeks later.

MK-3641 12 Amb a 1-U (short ragweed extract) and MK-7243 2800 BAU (timothy grass extract) were co-administered to patients [133]. Patients at least 18 years of age with ragweed and grass pollen-induced allergic rhinitis with or without conjunctivitis were recruited. After administration, the percentages of participants who experienced at least one event of local swelling were measured. During periods I, II, and III, they were 13.7%, 21.6%, and 14.7%, respectively. These results indicated that MK-3641 12 Amb a 1-U and MK-7243 2800 BAU are safe substances to treat AR.

The efficacy and safety of short ragweed pollen allergen extract sublingual immunotherapy tablets were assessed in children with ragweed-induced rhinoconjunctivitis with or without asthma [134]. The primary outcome was measured as the total combined score (TCS) during the peak ragweed season (RS). TCS is a daily symptom score (DSS) plus daily medication score (DMS), while RS means 15 consecutive days with the highest average pollen count. TCS could range from 0 to 38, and a lower score indicates fewer RC symptoms and medication use. TCS of short ragweed pollen allergen extract was 4.39 while the placebo was 7.12, thus implying short ragweed pollen allergen extract can be an effective treatment option for patients with rhinitis.

The optimal effective dose of SUBLIVAC FIX Birch was determined based on a decrease of upper airways reactivity after 5 months of treatment with different dosages of SUBLIVAC FIX Birch compared with placebo [135]. SUBLIVAC FIX Birch was administered in patients with allergic rhinitis/rhinoconjunctivitis caused by birch pollen. In all treatment groups, post-treatment symptom scores compared with baseline were improved. After active treatment, peak nasal inspiratory flow and serum IgG levels increased compared with placebo. All active dosages produced more adverse reactions than placebo, but those reactions were mainly mild and well-controlled.

Patients with allergic rhinitis/rhinoconjunctivitis caused by grass pollen were treated with SUBLIVAC FIX *Phleum pratense* [136]. This was a phase II, randomized, double-blind, and placebo-controlled study. The optimal effective dose of SUBLIVAC FIX Phleum pratense was determined with the same method as for SUBLIVAC FIX Birch. The dosages of five different *pratense* were 0, 3333, 10,000, 20,000, and 40,000 AUN/mL respectively.

After 10 months, a study about SUBLIVAC FIX Phleum Pratense for grass pollen-allergic patients with IgE-mediated seasonal allergic rhinoconjunctivitis (ARC) was conducted. [137]. The safety and tolerability of the *pratense* were assessed, and the dose-response signal was measured by using the total symptom score (TSS). The TSS of SUBLIVAC FIX Phleum Pratense (SP) 10,000, 40,000, and 80,000 AUN/mL were 8.06, 8.19, and 7.69 while the score of placebo was 10.01, demonstrating that SP could be effective in relieving total symptoms of ARC.

A trial tested cockroach subcutaneous immunotherapy (SCIT) safety in cockroach-sensitive adults with asthma and/or perennial allergic rhinitis. (ICAC-18) [138]. No severe adverse effects related to treatment were reported.

Mouse allergenic extract, called mouse epithelial extract or allergenic extract of Mus musculus, was administered by subcutaneous injection in mouse-sensitive adults with asthma or/and perennial allergic rhinitis (ICAC-26) [139]. The primary objective of this study was to assess whether treatment with mouse subcutaneous immunotherapy (SCIT) is safe. A total of nine adverse events (AEs) were reported, and no serious adverse events (SAEs) were reported. Although further studies are required to ensure the safety of mouse allergenic extract, perhaps the number of adverse events shows that the extract treats rhinitis safely.

## 4. Discussion

### 4.1. Mechanisms of Natural Products Regulating AR Symptoms

#### 4.1.1. T Helper Cell Differentiation

**Figure 2:**
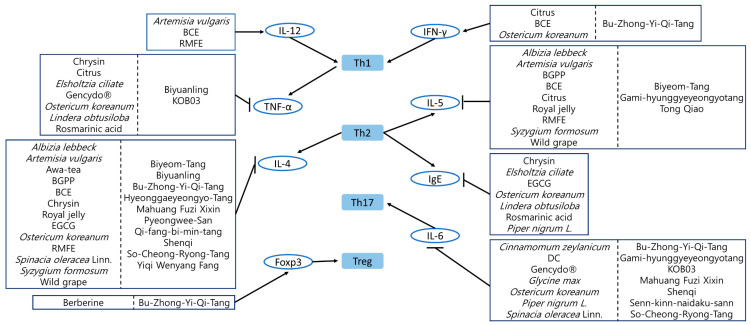
Schematic diagram of plants and decoctions that ameliorated allergic rhinitis by triggering T helper cell differentiation; BCE, *Bupleurum chinense* water extract; RMFE, Rosae Multiflorae Fructus water extract; BGPP, Brazilian green propolis; EGCG, Epigallocatechin gallate; DC, *Dryopteris crassirhizoma*.

Diverse studies referred to T helper cell differentiation when discussing the process of how the target extract works to treat allergic rhinitis (Figure 2). There were twenty five plant-originated natural products and fourteen decoctions modulating T helper cell differentiation.

IL-12, which is a Th1-related cytokine, was up-regulated by *Artemisia vulgaris* water extract, BCE, and RMFE [71,79,103]. Citrus, BCE, *Ostericum koreanum* root methanol extract, and Bu-Zhong-Yi-Qi-Tang showed their effects by increasing another Th1 cytokine, IFN-γ [60,66,79,117]. TNF-α is associated predominantly with Th1-mediated inflammation and is essential for the production of Th2 cytokines such as IL-4 and IL-12 [140]. The release of TNF-α was reduced by Chrysin, Citrus, *Elsholtzia ciliate* water extract, Gencydo^®^, *Ostericum koreanum* root methanol extract, *Lindera obtusiloba* water extract, Rosmarinic acid, Biyuanling, and KOB03 [58,60,61,63,64,66,68,116,122].

Th2 cytokines were the major route for regulation. Among plant-originated studies, *Albizia lebbeck* bark ethanol extract, *Artemisia vulgaris* water extract, Awa-tea leaf hot-water extract, BGPP, BGPP ethanol extract, BCE, Chrysin, Royal jelly, EGCG, *Ostericum koreanum* root methanol extract, RMFE, *Spinacia oleracea* Linn. aqueous extract, *Syzygium formosum* leaf extract, and Wild grape hot-water extract ameliorated allergic rhinitis by inhibiting IL-4 [55,56,57,58,66,69,71,72,79,86,103,104]. Among extract studies, Ethanol extract of Biyeom-Tang, Biyuanling, Bu-Zhong-Yi-Qi-Tang, Hyeonggaeyeongyo-Tang, Mahuang Fuzi Xixin decoction, Pyeongwee-San extract, Qi-fang-bi-min-tang, Shenqi, So-Cheong-Ryong-Tang, and Yiqi Wenyang Fang reduced IL-4 expression [114,115,116,117,118,124,125,126,128,131]. Furthermore, IL-5 was inhibited by *Albizia lebbeck* bark ethanol extract, *Artemisia vulgaris* water extract, BGPP, BGPP ethanol extract, BCE, Citrus, Royal jelly, RMFE, *Syzygium formosum* leaf extract, and Wild grape hot-water extract [55,56,57,60,71,72,79,103,104]. Ethanol extract of Biyeom-Tang, Gami-hyunggyeyeongyotang, and Tong Qiao nose drop also suppressed expression of IL-5 [111,115,129]. There were 7 plant extracts and 7 decoctions that modulated IgE level. *Cinnamomum zeylanicum* bark hydroalcoholic extract, DC, Gencydo^®^, *Glycine max* water extract, *Ostericum koreanum* root methanol extract, *Piper nigrum* L. extract, and *Spinacia oleracea* Linn. aqueous extract down-regulated IgE levels [63,66,83,85,89,93,104]. Bu-Zhong-Yi-Qi-Tang, Gami-hyunggyeyeongyotang, KOB03, Mahuang Fuzi Xixin decoction, Shenqi, Senn-kinn-naidaku-sann, and So-Cheong-Ryong-Tang prohibited IgE production [111,113,114,117,122,124,128].

Th17-related cytokine IL-6 was suppressed by seven plant extracts. Chrysin, *Elsholtzia ciliate* water extract, EGCG, *Ostericum koreanum* root methanol extract, *Lindera obtusiloba* water extract, Rosmarinic acid, and *Piper nigrum* L. extract mediated allergic reactions by down-regulating IL-6 [58,61,64,66,68,86,93].

Berberine and Bu-Zhong-Yi-Qi-Tang increased Foxp3, which transits Th0 cells to Treg cells and ameliorates allergic symptoms [77,117].

#### 4.1.2. Histamine Receptors

**Figure 3:**
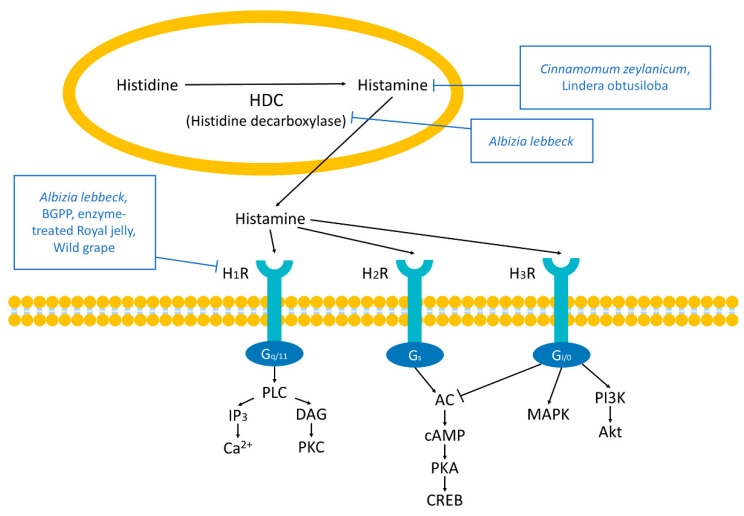
Histamine receptors. HDC, histidine decarboxylase; H1R, histamine H1 receptor; H_2_R, histamine H2 receptor; H_3_R, histamine H3 receptor; G, G protein; IP_3_, inositol 3,4,5-triphosphate; DAG, diacylglycerol; PKC, protein kinase C; AC, adenylyl cyclase; cAMP, cycling AMP; PKA, protein kinase A; CREB, cAMP-responsive element binding protein; MAPK, mitogen-activated protein kinase; Pl3K, phosphatidylinositol 3-kinase; Akt, protein kinase B; BGPP, Brazilian green propolis.

There were six plant-originated studies discussing histamine receptor functions (Figure 3). H1R was down-regulated by *Albizia lebbeck* bark ethanol extract, BGPP, enzyme-treated Royal jelly, and wild grape [55,56,72]. *Cinnamomum zeylanicum* bark hydroalcoholic extract and *Lindera obtusiloba* water extract reduced histamine release [64,83]. *Albizia lebbeck* bark ethanol extract regulated histidine decarboxylase [55].

#### 4.1.3. 5-Lipooxygenase Pathway and Prostaglandin E2 Synthesis-and-Signaling Pathway

**Figure 4:**
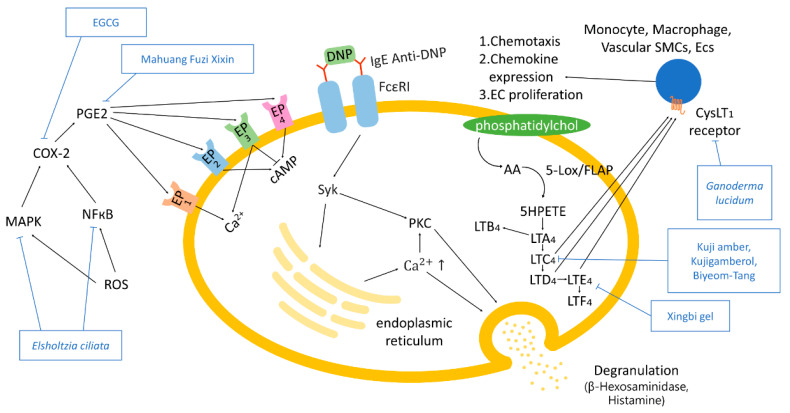
LO pathway, PGE_2_ synthesis and signaling pathway. MAPK, mitogen-activated protein kinase; COX-2, cyclooxygenase-2; NFκB, nuclear factor-κB; ROS, reactive oxygen species; PGE_2_, prostaglandin E2; DNP, dinitrophenyl; IgE, immunoglobulin E; FcεRI, high-affinity IgE receptor; Syk, spleen tyrosine kinase; PKC, protein kinase C; AA, arachidonic acid; 5-Lox, arachidonate 5-lipoxygenase; FLAP, 5-LO-activating protein; 5HPETE, 5-hydroperoxyeicosatetraenoic acid; LT, leukotriene; EC, endothelial cell; SMC, smooth muscle cell; CysLT1, cysteinyl leukotriene 1; EGCG, Epigallocatechin gallate.

Two plant-originated natural products, three minerals, and three decoction studies discussed the 5-lipooxygenase pathway (Figure 4). MAPK and NF-κB were suppressed by *Elsholtzia ciliata* water extract [61]. EGCG regulated COX-2, thus reducing allergic symptoms [86]. CysLT1 receptor activation was down-regulated by *Ganoderma lucidum* powder [110]. LTC_4_ was suppressed by Kuji amber, Kujigamberol, and ethanol extract of Biyeom-Tang [106,115]. Mahuang Fuzi Xixin decreased PGE_2_, and Xingbi gel regulated the secretion of LTE_4_ [112,130].

#### 4.1.4. Immune Response Pathways

**Figure 5:**
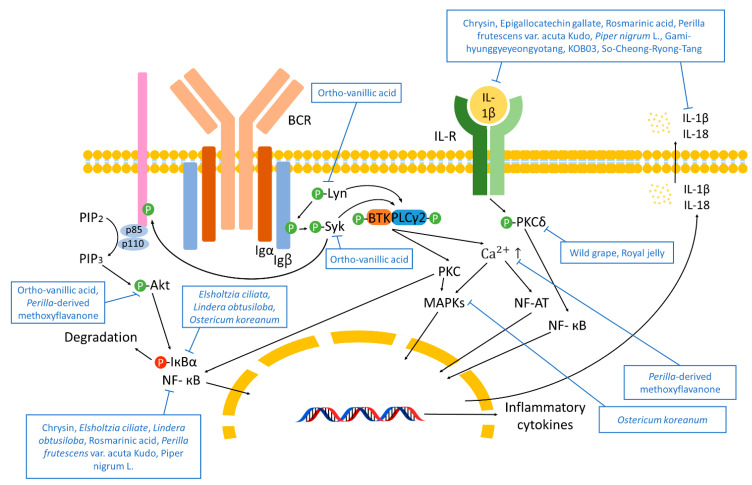
Various pathways for immune response. PIP_2_, phosphatidyl inositol 4, 5-trisphosphate; PIP_3_, phosphatidylinositol 3, 4, 5-trisphosphate; P, phosphate; NF-κB, p65 nuclear factor kappa B; BCR, B cell receptor; Ig, immunoglobulin; Syk, spleen tyrosine kinase; BKT, Bruton’s tyrosine kinase; PLCγ2, phospholipase C gamma 2; IL, interleukin; IL-R, interleukin receptor; MAPK, mitogen-activated protein kinase; NF-AT, nuclear factor of activated T-cells.

There were eleven studies regarding plant-originated natural products and three decoction studies discussing histamine receptors’ functions in immune responses (Figure 5). p-Akt was regulated by Ortho-vanillic acid and *Perilla*-derived methoxyflavanone [65,67]. NF-κB was inhibited by Chrysin, *Elsholtzia ciliata* water extract, *Lindera obtusiloba* water extract, Rosmarinic acid, Perilla frutescens var. acuta Kudo 30% ethanol extract powder, and Piper nigrum L. extract [58,61,64,68,93]. p-IκBα was reduced by *Elsholtzia ciliata* water extract, *Lindera obtusiloba* water extract, and *Ostericum koreanum* root methanol extract [61,64,66]. p-Lyn and p-Syk are regulated by Ortho-vanillic acid [65]. The treatment of *Ostericum koreanum* root methanol extract suppressed MAPKs [66]. Perilla frutescens var. acuta Kudo 30% ethanol extract powder ameliorated allergic inflammatory reactions by decreasing Ca^2+^ [68]. Enzyme-treated Royal jelly and wild grape hot-water extract inhibited the expression of p-PKCδ [56,72]. Chrysin, Epigallocatechin gallate, Rosmarinic acid, Perilla frutescens var. acuta Kudo 30% ethanol extract powder, Piper nigrum L. extract, Gami-hyunggyeyeongyotang, KOB03, and So-Cheong-Ryong-Tang regulated IL-1β [58,68,86,93,118,122,127].

### 4.2. Comparison Analysis

Some compounds or extracts were examined in more than one study. There were two different studies about BGPP. The studies differed in experimental subjects, dose, duration, mechanism, and efficacy of the extract. Shaha et al. used HeLa cells, RBL-2H3 cells, and a TDI-sensitized rat model, while Tani et al. used Cry j1-treated PBMCs and Cry j1-treated peripheral leukocytes of allergic patients [56,57]. The former study showed the relationship between improvement of nasal symptoms and degradation of H1R mRNA, p-PKCδ, IL-9, IL-4, and IL-5; the latter explained that nasal obstruction preventing property of BGPP was related to degradation of IL-5, IL-14, and CysLTs. Two different studies examined the extract of Piper nigrum L. (PNE) as AR treatment in vivo [90,93]. Bui et al. and Bang et al. both used OVA-induced AR mice, but the species differed—BALB/c mice and Swiss albino mice, respectively. The former administered 50, 100, and 200 mg/kg of PNE and the latter 10, 20, and 40 mg/kg. Bui et al. compared the efficacy of PNE with that of Dex, while Bang et al. did so with montelukast. Different mechanisms related to AR symptoms were investigated: the former focused on the inhibition of STAT3 and NFκBp65 plus the elevation of Th1, whereas the latter focused on the inhibition of IL-6, IL-1β, and IgE. KOB03 was examined by various methods [122,141]. OVA-induced AR BALB/c mice and SD rats were administered with 100 or 200 mg/kg of KOB03 for 7 days. The efficacy of the decoction was generated by inhibiting TNF-α, IL-1β, IL-6, and IL-8. In comparison, only OVA-induced AR BALB/c mice were administered with a higher dose, 5 mg/kg, for 8 days. As a result, the decrease of IgE, LTC_4_, IL-4, and IL-1β inhibited nasal inflammation. Ren et al. and Tang et al. examined the efficacy of the Mahuang Fuzi Xixin Decoction respectively [112,124]. Ren et al. conducted in vivo experiment using OVA-induced AR SPF Wi star rats, while Tang et al. organized an in vitro experiment. Ku et al. and Mo et al. experimented with different doses and durations of So-Cheong-Ryong-Tang: 20 mg/mL for 14 days and 1 g/kg for 7 days, respectively [127,128].

### 4.3. Limitations

The reviewed studies had some limitations. There were experiments with high doses (over 60 μg/mL in vitro) of the compound or extract [56,57,61,62,111,114]. Some experiments were conducted only in in vitro [57,58,60,61,62,63,66,67,70,106]. The nasal symptoms and pathogenesis observed in the studies using the TDI-sensitized rhinitis model may differ from AR, an IgE mediated disease, as TDI-sensitized rhinitis is a non-IgE mediated disease [55,56,72,89,116,142]. Furthermore, this paper had shortcomings that should be remedied in the following studies. The papers are predominantly about compounds and extracts that originated from plants. Clinical trials lack quantity compared with in vivo and in vitro experiments, eight to a total of forty-nine. Additionally, this review was limited to studies published in the last 10 years and written in English. Above all, although a variety of compounds and extracts were included, examination for each of them was insufficient.

### 4.4. Significance

This review included specific mechanisms of AR and its treatment, examined the result of diverse research, and contains specific figures of AR mechanisms. As various compounds and extracts were reviewed, this paper details the potential for the widespread use of natural products in treating AR. Further studies about toxicity, stability, and pharmacokinetics based on this review could be conducted to confirm their possibilities.

## 5. Methods

Relevant articles published between 2009 and 2019 regarding the therapeutic effect of plant extracts on allergic rhinitis were collected from PUBMED, the Google Scholar database, and the Web of Science. The search algorithm was designed by entering related keywords such as ‘allergic rhinitis’, ‘natural product’, ‘herbal medicine’, ‘decoction’, ‘tang’, ‘extract’, and ‘clinical trial’. We only reviewed articles written in English and excluded duplicates and studies with English abstracts but non-English articles. The papers were first classified into two categories based on the form of the experimental model: in vivo and in vitro. Then, we reclassified in vitro studies into three categories according to the kinds of natural resources used in the experiment: plant, etc., and decoction. “Etc.” includes animal and fungi, which were very few in number to be exclusively classified. For data unity and a better understanding of the influence of compounds on allergic rhinitis, we only considered single compounds except for decoction. Decoctions were included for the inclusiveness of the study; plant extracts are frequently used in clinical situations in the form of decoction or tang. Overall, fifty-seven studies demonstrating the efficacy of using plant extracts in treating nasal inflammation were reviewed.

## 6. Conclusions

Natural products can be used as an effective treatment for AR. In most of the studies reviewed, they showed positive effects on relieving the symptoms of AR, such as rubbing of the nose and sneezing, or inhibiting inflammation in vivo and in vitro. The results of clinical trials showed that the treatments were effective and safe. Therefore, natural products could be attractive candidates for drug development for treating AR.

## Figures and Tables

**Table 3:** In vitro studies—fungi and minerals-originated natural products.

Classification	Compound	Source	Cell Line/Animal Model	Dose/Duration	Efficacy	Mechanism	Reference
Mineral	Kuji amber methanol extract	Succinum	RBL-2H3	2 μL (0–25 μg/mL); 30 min	Inhibition of the degranulation of RBL-2H3 cells	↓ p-ERK1/2, LTC_4_	[106]
Mineral	Kujigamberol (15,20-dinor-5,7,9-labdatrien-18-ol)	Succinum	RBL-2H3	2 μL (0–25 μg/mL); 30 min	Inhibition of the degranulation of RBL-2H3 cells	↓ p-ERK1/2, LTC_4_	[106]

p-ERK1/2, phospho-extracellular signal-regulated kinase1/2; LTC_4_, Leukotriene C4; ↓, down-regulation.

**Table 4:** In vivo studies—Fungi and minerals.

Classification	Compound	Source	Cell Line/Animal Model	Dose/Duration	Efficacy	Mechanism	Reference
Fungi	*Ganoderma lucidum* powder suspended in water	*Ganoderma lucidum*	Cedar pollen extracts-sensitized AR guinea pigs	100, 1000 mg/5 mL/kg; 8 weeks	Inhibition of nasal inflammation	↓ CysLT1	[110]
Mineral	Kuji amber methanol extract	Succinum	OVA-induced rhinitis guinea pigs	20 μg; 10 min, 1 h	Inhibition of nasal blockade	↓ p-ERK1/2, LTC_4_	[106]
Mineral	Kujigamberol (15,20-dinor-5,7,9-labdatrien-18-ol)	Succinum	OVA-induced rhinitis guinea pigs	20 μg; 10 min, 1 h	Inhibition of nasal blockade	↓ p-ERK1/2, LTC_4_	[106]

CysLT1, Cysteinyl leukotriene receptor 1; OVA, ovalbumin; p-ERK1/2, phospho-extracellular signal-regulated kinase1/2; LTC_4_, Leukotriene C4; ↓, down-regulation.

**Table 5:** In vitro studies—decoctions of natural products.

Name	Constitutions	Experimental Subject	Dose; Duration	Efficacy	Mechanism	Reference
Gami-hyunggyeyeongyotang	Schizonepetae Spica (*Nepeta tenuifolia* Benth.), Forsythiae Fructus (*Forsythia viridissima* Lindl.), Saposhnikoviae Radix (*Saposhnikovia divaricata* (Turcz.) Schischk.), Angelicae Radix (*Angelica acutiloba* Kitagawa), Cnidii Rhizoma (*Cnidium officinale* Makino), Paeoniae Radix Alba (*Paeonia lactiflora* Pallas), Angelicae Dahuricae Radix (*Angelica dahurica Bentham* et Hooker), Bupleuri Radix (*Bupleurum falcatum* Linne), Scutellariae Radix (*Scutellaria baicalensis* Georgi), Aurantii Fructus Immaturus (*Citrus aurantium* Linne), Gardeniae Frutus (*Gardenia jasminoides* Ellis), Platycodi Radix (*Platycodon grandiflorum* A. De candole), Glycyrrhizae Radix (*Glycyrrhiza uralensis* Fischer), Ulmi Cortex (*Ulmus macrocarpa* Hance), Xanthii Fructus (*Xanthium strumarium* Linne), Magnoliae Flos (*Magnolia kobus* De Candolle), Rubiae Radix (*Rubia akane* Nakai)	HMC-1	1, 10, 100 μg/mL; 1 h	Alleviation of the symptoms of AR	↓ IgE, IL-5, IL-6, IL-1β, MCP-1, MIP-2, caspase-1	[111]
Mahuang Fuzi Xixin Decoction	Ephedrae Herba (*Ephedra sinica* Stapf, *Ephedra intermedia* Schrenk & C.A. Mey. or *Ephedra equisetina* Bunge), Aconiti Lateralis Radix (*Aconitum carmichaelii* Debeaux), Asiasari Radix (*Asarum heterotropoides* f. *mandshuricum* (Maxim.) Kitag.)	IgE-activated RBL-2H3	M136: 3, 10, 30 μM; 1 h	Anti-inflammatory effect	↓ PGE2	[112]
			M188: 5, 10 μM; 1 h			
			M234: 2, 5 μM; 1 h			
			M289: 3, 10, 30 μM; 1 h			
Senn-kinn-naidaku-sann	Ginseng Radix, Angelicae Radix, Astragali Radix, Cnidii Rhizoma, Sinomeni Caulis et Rhizoma, Platycodonis Radix, Magnoliae Cortex, Angelicae Dahuricae Radix, Glycyrrhizae Radix	RAW264.7	0.01, 0.1, 1, 10 μg/mL; 24 h	Inhibition of inflammatory response	↑ TLR4, IL-12	[113]
Shenqi	Angelicae Dahuricae Radix (*Angelica dahurica* (Hoffm.) Benth. & Hook.f. ex Franch. & Sav.)*,* Scutellariae Radix (*Scutellaria baicalensis* Georgi)*,* Lonicerae Japonicae Flos (*Lonicera japonica* Thunb.)*,* Menthae Haplocalycis Herba (*Mentha canadensis* L.), Astragali Radix (*Astragalus propinquus* Schischkin)*,* Codonopsis Radix (*Codonopsis pilosula* subsp. *tangshen* (Oliv.) D.Y.Hong)*,* Isatidis Folium (*Isatis tinctoria* L.)*,* Taraxaci Herba (*Taraxacum mongolicum* Hand.-Mazz.)*,* Agrimoniae Herba (*Agrimonia pilosa* Ledeb.), Asiasari Radix et Rhizome (*Asarum sieboldii* Miq.)	RBL-2H	50, 100, 200 μg/mL; 1 h	Alleviation of symptoms of AR	↓ β-Hex, histamine	[114]

AR, allergic rhinitis; β-Hex, β-hexosaminidase; IL-5, interleukin 5; IgE, immunoglobulin E; HMC-1, human mast cell line; IL-6, interleukin 6; IL-1β, interleukin 1 beta; MCP-1, monocyte chemoattractant protein; MIP-2, macrophage inflammatory protein; STAT6, signal transducer and activator of transcription 6; RBL-2H3, rat basophilic leukemia; PGE2, prostaglandin E2; TLR4, Toll-like receptor 4; IL-12, interleukin 12; ↑, up-regulation; ↓, down-regulation.

**Table 7:** Clinical trials.

Classification	Compound/Extract	Source	Phase	Patients	Status	Registration Number	Reference
Plant	Depigoid 50% Grasses/50% *Olea europaea* (2000DPP/mL), Depigoid 50% Grasses/50% *Parietaria judaica* (2000DPP/mL)	Grasses, *Olea europaea* L., *Parietaria judaica* L.	Phase 2	63	Completed	NCT01734265	[132]
Plant	MK-3641 12 Amb a 1-U (short ragweed extract) MK-7243 2800 BAU (Timothy grass extract)	*Ambrosia artemisiifolia* L., *Phleum pratense* L.	Phase 4	102	Completed	NCT02256553	[133]
Plant	Short ragweed pollen allergen extract	*Ambrosia artemisiifolia* L.	Phase 3	1025	Completed	NCT02478398	[134]
Plant	SUBLIVAC FIX Birch	*Betula verrucose* Ehrh.	Phase 2	269	Completed	NCT01639768	[135]
Plant	SUBLIVAC FIX *Phleum pratense*	*Phleum pratense* L.	Phase 2	266	Completed	NCT01682070	[136]
Plant	SUBLIVAC FIX *Phleum Pratense*	*Phleum pratense* L.	Phase 2	168	Completed	NCT02556801	[137]
Animal	German cockroach (*Blattella germanica*) allergenic extract	*Blattella germanica*	Phase 1	10	Completed	NCT01221285	[138]
Animal	Mouse Allergenic Extract	*Mus musculus*	Phase 1 Phase 2	12	Completed	NCT02532179	[139]

## Data Availability

Not applicable.
